# Biosensors for the detection of chorismate and *cis,cis*-muconic acid in *Corynebacterium glutamicum*

**DOI:** 10.1093/jimb/kuae024

**Published:** 2024-06-29

**Authors:** Jeanette C Velasquez-Guzman, Herbert M Huttanus, Demosthenes P Morales, Tara S Werner, Austin L Carroll, Adam M Guss, Chris M Yeager, Taraka Dale, Ramesh K Jha

**Affiliations:** Bioscience Division, Los Alamos National Laboratory, Los Alamos, NM 87545, USA; Chemistry Division, Los Alamos National Laboratory, Los Alamos, NM 87545, USA; Agile BioFoundry, Emeryville, CA 94608, USA; Bioscience Division, Los Alamos National Laboratory, Los Alamos, NM 87545, USA; Agile BioFoundry, Emeryville, CA 94608, USA; Center for Integrated Nanotechnologies, Los Alamos National Laboratory, Los Alamos, NM 87545, USA; Bioscience Division, Los Alamos National Laboratory, Los Alamos, NM 87545, USA; Agile BioFoundry, Emeryville, CA 94608, USA; Agile BioFoundry, Emeryville, CA 94608, USA; Biosciences Division, Oak Ridge National Laboratory, Oak Ridge, TN 37830, USA; Agile BioFoundry, Emeryville, CA 94608, USA; Biosciences Division, Oak Ridge National Laboratory, Oak Ridge, TN 37830, USA; Chemistry Division, Los Alamos National Laboratory, Los Alamos, NM 87545, USA; Agile BioFoundry, Emeryville, CA 94608, USA; Bioscience Division, Los Alamos National Laboratory, Los Alamos, NM 87545, USA; Agile BioFoundry, Emeryville, CA 94608, USA; Bioscience Division, Los Alamos National Laboratory, Los Alamos, NM 87545, USA; Agile BioFoundry, Emeryville, CA 94608, USA

**Keywords:** Promoter optimization, Flow cytometry, High-throughput screening, Biosensor, *cis,cis*-muconic acid, Chorismate, Biomanufacturing, Tool transfer, Non-model organism

## Abstract

*Corynebacterium glutamicum* ATCC 13032 is a promising microbial chassis for industrial production of valuable compounds, including aromatic amino acids derived from the shikimate pathway. In this work, we developed two whole-cell, transcription factor based fluorescent biosensors to track *cis,cis*-muconic acid (ccMA) and chorismate in *C. glutamicum*. Chorismate is a key intermediate in the shikimate pathway from which value-added chemicals can be produced, and a shunt from the shikimate pathway can divert carbon to ccMA, a high value chemical. We transferred a ccMA-inducible transcription factor, CatM, from *Acinetobacter baylyi* ADP1 into *C. glutamicum* and screened a promoter library to isolate variants with high sensitivity and dynamic range to ccMA by providing benzoate, which is converted to ccMA intracellularly. The biosensor also detected exogenously supplied ccMA, suggesting the presence of a putative ccMA transporter in *C. glutamicum*, though the external ccMA concentration threshold to elicit a response was 100-fold higher than the concentration of benzoate required to do so through intracellular ccMA production. We then developed a chorismate biosensor, in which a chorismate inducible promoter regulated by natively expressed QsuR was optimized to exhibit a dose-dependent response to exogenously supplemented quinate (a chorismate precursor). A chorismate–pyruvate lyase encoding gene, *ubiC*, was introduced into *C. glutamicum* to lower the intracellular chorismate pool, which resulted in loss of dose dependence to quinate. Further, a knockout strain that blocked the conversion of quinate to chorismate also resulted in absence of dose dependence to quinate, validating that the chorismate biosensor is specific to intracellular chorismate pool. The ccMA and chorismate biosensors were dually inserted into *C. glutamicum* to simultaneously detect intracellularly produced chorismate and ccMA. Biosensors, such as those developed in this study, can be applied in *C. glutamicum* for multiplex sensing to expedite pathway design and optimization through metabolic engineering in this promising chassis organism.

**One-Sentence Summary:**

High-throughput screening of promoter libraries in *Corynebacterium glutamicum* to establish transcription factor based biosensors for key metabolic intermediates in shikimate and β-ketoadipate pathways.

## Introduction

Nature has a vast variety of microorganisms with unique phenotypes fully adapted to survive in extreme conditions and exhibiting unique and versatile metabolisms that are attractive as potential industrial platforms for bioconversion (Calero & Nikel, 2019). Despite their versatility, these microorganisms often require development and implementation of genetic engineering tools for their “domestication” to enable their productive use as industrial strains (Riley & Guss, 2021). One such organism is *Corynebacterium glutamicum* ATCC 13032 (hereafter referred to as *C. glutamicum*), a non-pathogenic, Gram-positive soil bacterium that is a promising candidate for production of industrially relevant chemicals, including biofuels, cosmetics, pharmaceuticals, and a wide range of acids that include amino acids and various other organic acids (Becker, Rohles et al., 2018; Inui & Toyoda, 2020; Lin et al., 2022). To realize the full potential of this organism as an industrial chassis for the production of biochemicals, advancement in metabolic engineering tools such as genome editing (Wang et al., 2021) and high-throughput screening (described below) are needed.

Biosensors have gained substantial interest in metabolic engineering (Kaczmarek & Prather, 2021; Mahr & Frunzke, 2016) for screening libraries of microbial strains, dynamic regulation of metabolic pathways, single-cell analysis, and screening of microbial populations generated from adaptive laboratory evolution (Bentley et al., 2020; Seok et al., 2021; Zhang et al., 2012). Whole-cell biosensors usually contain a sensor–reporter gene circuit, which responds to the presence of the molecule of interest by generating a survival, bioluminescent, colorimetric, or fluorescent response. Existing biosensors for *C. glutamicum* are mainly comprised of native transcription factors (TFs) that detect and respond to small molecule targets and have been applied for high-throughput screening of various libraries generated rationally or through adaptive laboratory evolution (Han et al., 2020; Mahr et al., 2015; Schendzielorz et al., 2014; Schulte et al., 2017; Siedler et al., 2017; Sonntag et al., 2020; Steffen et al., 2016). Recently, a functional shikimate biosensor was constructed from a LysR-type transcriptional regulator ShiR, a native TF from *C. glutamicum* that exhibited a detection range of 1–100 mM and was used to monitor shikimate production and to optimize ribosome binding sites to increase the carbon flux in the shikimate pathway (Liu et al., 2018).

**Fig. 1.**
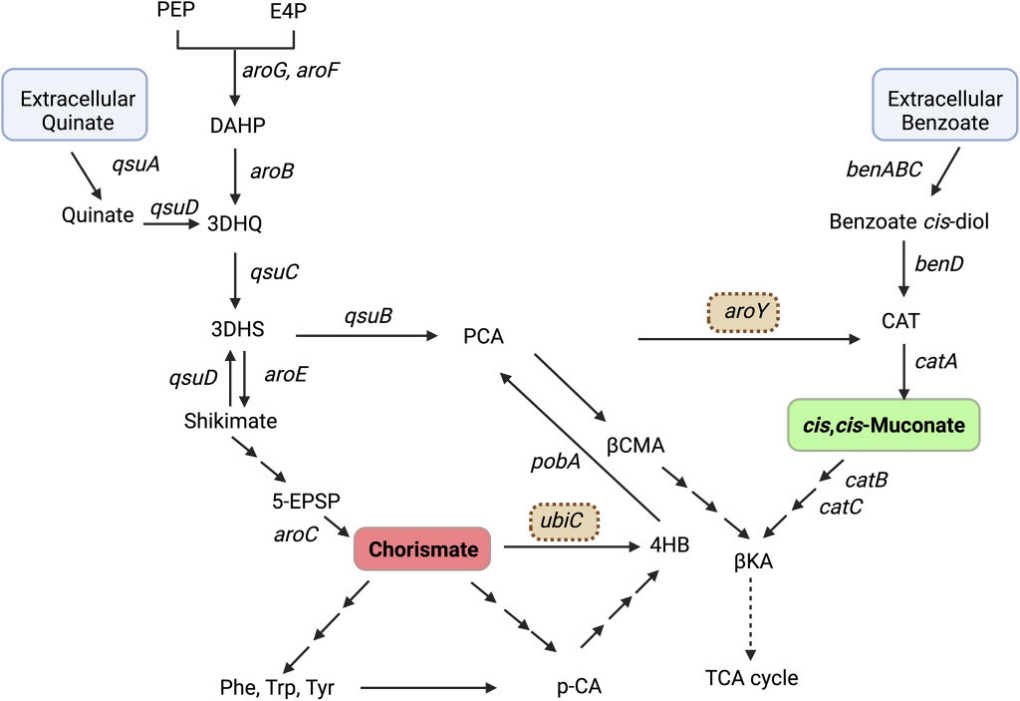
A schematic of the shikimate and β-ketoadipate pathways. In the *C. glutamicum* strain used in this study, the shikimate and β-ketoadipate pathways are connected by the native *qsuB* encoding 3-dehydroshikimate dehydratase (DHSase), as well as by heterologous enzymes, including chorismate pyruvate-lyase (UbiC, which converts chorismate to 4-hydoxybenzoate) and PCA decarboxylase (AroY, which converts PCA to catechol). Two of the industrially relevant molecules in these pathways are chorismate from the shikimate pathway and *cis, cis*-muconate (ccMA) from the β-ketoadipate pathway. Abbreviations: PEP, Phosphoenolpyruvate; E4P, Erythrose 4-phosphate; DAHP, 3-deoxy-D-arabino-heptulosonate 7-phosphate; 3DHQ, 3-Dehydroquinate; 3DHS, 3-Dehydroshikimate; 5-EPSP, 5-Enolpyruvylshikimate 3-phosphate; 4HB, 4-hydroxybenzoate; PCA, Protocatechuate; p-CA, p-Coumarate; βCMA, β-Carboxymuconate; βKA, β-ketoadipate; TCA, Tricarboxylic acid; CAT, Catechol. Created with BioRender.com

In this work, we focused on the development and implementation of two TF-based fluorescent biosensors in *C. glutamicum* targeting metabolic intermediates in the shikimate and β-ketoadipate pathways (Fig. 1), with the aim of adding to the synthetic biology toolset for this highly promising microbial chassis. Firstly, we established a *cis,cis*-muconic acid (ccMA) biosensor *in C. glutamicum* using CatM, a LysR-type transcription regulator (LTTR) (Maddocks & Oyston, 2008) from the soil bacterium *Acinetobacter baylyi* ADP1 (Ezezika et al., 2006). CatM responds to the effector molecule ccMA, and the experimentally determined structure of CatM confirms ccMA bound to CatM in the interdomain pocket (Ezezika et al., 2007). The CatM promoter was previously optimized for a ccMA biosensor in *Pseudomonas putida* by screening a CatM regulated promoter library and was shown to respond specifically to ccMA (Bentley et al., 2020). That same promoter library was used in this study and screened for biosensing performance in *C. glutamicum*. Secondly, we targeted the detection of chorismate, which is the final product of the shikimate pathway and a key intermediate for the synthesis of a wide range of aromatic compounds, including aromatic amino acids as well as various coenzymes (Averesch & Krömer, 2018; Masuo et al., 2016). By utilizing a native chorismate-dependent transcription regulator QsuR (Kubota et al., 2014) and screening a diversified *P_qsu_* promoter regulating a fluorescent reporter, we established a chorismate biosensor with improved sensitivity and dynamic range in *C. glutamicum* when compared to the simpler strategy of combining the native *P_qsu_* promoter with the same fluorescent reporter. While the biosensors are not precise enough to replace more quantitative tools like high-performance liquid chromatography (HPLC) (for extracellular titer of a metabolite) and mass spectrometry (MS)-based metabolomics (for intracellular metabolite concentration), the utility of theses biosensors spans from estimating relative carbon flux through their respective metabolic nodes to high-throughput screening of strains for productivity. Considering that the conversion of glucose to ccMA results from diversion of carbon from the shikimate pathway, dual sensing of chorismate and ccMA will be an attractive approach for estimating and optimizing the distribution of carbon flux between the shikimate pathway and the shunt pathway, respectively.

## Material and Methods

### Plasmid Construction

For plasmid construction, fragments were assembled by NEBuilder HiFi Assembly Master Mix (New England Biolabs) followed by transformation into MAX Efficiency™ DH5α *Escherichia coli* competent cells (Thermo Fisher Scientific) or *C. glutamicum* ATCC 13032 competent cells (described below). The shuttle vector backbone and the biosensor–reporter cassette were amplified by PCR.

### Construction of a Diversified Promoter Library for *cis, cis-*Muconate Biosensor

ccMA biosensor construction was initially done in pBL1/ColE1(pLFC007) shuttle vector with enhanced Green Fluorescent Protein (eGFP) reporter expressed by the *P_BAD_* promoter. Later, the pBL1/ColE1vector was modified by inserting the CatM gene from *A. baylyi* ADP1 with promoter *P_cat_* and superfolder GFP as reporter described before in a previous work (Bentley et al., 2020). The sensor pCatM_C2 was ported into *C. glutamicum*. The primers are listed in Table S1. The *P_cat_* was optimized with library diversification and mutation in the −35 and −10 site and CatM operator site 3, ribosome binding site (RBS), and spacer. The biosensor plasmid with optimized promoter was named pRJ2010 and the *C. glutamicum* Δ*catB* strain harboring the plasmid was named RJ95A.

### Construction of a Diversified Promoter Library for Chorismate Biosensor

The chorismate biosensor plasmid was constructed with QsuR promoter (*P_qsu_*) controlling a Cherry fluorescent protein (mCherry) as a reporter. The primers used for diversification of the QsuR promoters are listed in Supplementary Information 2. The *P_qsu_* library includes diversification of the −35 region, −10 region, and both ends of the putative third QsuR binding site (site 3). The PCR fragments were assembled using overlap extension PCR to generate a promoter library with a theoretical diversity of above 260 000. The plasmid library consisting of the diversified promoter was named pJV5.

### Transformation of Plasmid Constructs in *C. Glutamicum* Strains

Preparation of *C. glutamicum* competent cells and plasmid transformation were performed as described previously (Ruan et al., 2015). An overnight culture of *C. glutamicum* was grown in 5 mL BHIS (Brain heart infusion (BHI) broth supplemented with 91.1 g/L sorbitol) and then inoculated into 50 mL of NCM (each liter of NCM consists of 17.4 g K_2_HPO_4_, 11.6 g NaCl, 5 g glucose, 5 g tryptone, 1 g yeast extract, 0.2 g trisodium citrate, 0.05 g MgSO_4_.7H_2_O, 91.1 g sorbitol, 3 g glycine, 0.4 % isoniazid (INH), and 0.1 % Tween 80 and adjusted to pH 7.2 with 1 M NaOH) and grown for 3–6 hr to an optical density at a wavelength of 600 nM (OD_600_) of 1. The culture was chilled on ice for 10 min, split into 25 mL aliquots in two 50 mL centrifuge tubes, and 25 mL of 10 % ice cold glycerol was added, followed by centrifugation at 3500 × *g* for 10 min at 4°C. The cells were washed two times with 100 mL of ice cold 10% glycerol. The supernatant was removed, and the cells were resuspended in 200 μL of ice cold 10% glycerol and 80 μL aliquots of competent cells were stored at −80°C. For transformation, cells were thawed on ice and 100 ng of replicative plasmid DNA was added to the competent cells. The cells were moved to a 0.2 cm electroporation cuvette and electroporation at 2.5 kV, 200 Ω, and 25 μF was performed. After transformation, 920 μL of BHIS medium was added to the electroporation cuvette, then the electroporated cells were moved into a 1.5 mL microcentrifuge tube and cells were heat shocked at 46°C for 6 min. For recovery, transformed cells were grown at 30°C for 2–4 hr. After that the cells were centrifuged at 6000 rpm for 5 min, 800 μL of supernatant was removed and 200 μL was dispensed onto the LBHIS agar plates (5 g/L tryptone, 5 g NaCl, 2.5 g yeast extract, 18.5 g/L BHI, 91 g sorbitol, and 18 g agar and adjusted to pH 7.2 with 1 M NaOH) plates and cells were grown at 30°C for 1–2 days until colonies appear.

### Construction of Dual Sensing in *C. Glutamicum*

To build a dual sensor, chorismate biosensor plasmid (pJV5E.2) was used as a backbone and was digested with KpnI to introduce *catM-P_cat-_opt-2-sfgfp* muconate sensing cassette. pRJ2010 harboring muconate cassette was used as a template for PCR amplification. In addition, *soxR* and *tonB* terminator sequences were introduced around *catM* gene and *P_qsu_*. Primers used in this construction are listed in Supplementary Information 2. The new construct carrying two biosensors was named as pJV8.

### Construction of a Temperature Sensitive (Ts) Muconate Biosensor for *C. Glutamicum*

To create a Ts muconate biosensor, the muconate sensor-reporter cassette from pRJ2010 was inserted into a plasmid pALC412 (synthesized by GenScript), which contains a temperature sensitive replicase (RepA) and an apramycin resistance gene (ApmR) marker. The mutation in RepA protein was described before with an amino acid substitution of proline to serine at amino acid 47 (P47S) (Nakamura et al., 2006). The plasmid also contain pMB1 and *bla* gene for maintenance in *E. coli.* The resulting chimeric construct was named JV4 and transformed into *C. glutamicum* Δ*catB* competent cells. The plasmid curing assay was done at 30°C and 37°C with and without apramycin (12 μg/mL). To start the culture, one scoop from a JV4 glycerol stock was inoculated in 5 mL of BHIS (± apramycin). The next day, the culture was diluted 100-fold in BHIS (first passage) and after 24 hr of incubation the cell sample was diluted again in fresh BHIS (Second passage) with and without antibiotics. When cells reached an OD_600_ of 0.6, cells were induced with 0.5 mM catechol for overnight induction. Next day, JV4 cells were diluted in PBS to measure fluorescence intensity in Accuri C6 Plus Flow Cytometer BD C sampler™ Plus.

### Cell Growth Condition and Induction

Small scoops of glycerol stock of *C. glutamicum* biosensor strains were inoculated in 3 mL of BHIS with 10 μg/mL of chloramphenicol or 12 ug/mL of Apramycin and grown overnight at 30°C with shaking at 250 rpm. Next day cells were diluted 100-fold in fresh growth media and grown for 4–5 hr until an OD_600_ of ∼0.6. The cell cultures were then split into aliquots of 180 μL each in 96 deep well plates with 20 μL of inducers and allowed to grow at 30°C in a plate shaker at 1000 rpm for ∼16 hr. For the ccMA biosensor, cells were grown in BHIS induced with 0, 0.1, 1, 3, and 10 mM of ccMA or ccMA precursor (benzoate, catechol and protocatechuic acid (PCA). Cells carrying a chorismate biosensor were induced with 0, 0.1, 1, 3, and 10 mM of quinate.

### Measurement of Fluorescence Intensity of *C. glutamicum* Strains

For measuring fluorescence intensity, 20 μL of cell samples from overnight culture were diluted into 180 μl of 1× phosphate buffer saline (PBS) and placed into 96 well plates and analyzed in a microplate reader Tecan Infinite M200 at room temperature. Superfolder green fluorescent protein (sfGFP) fluorescence was determined using an excitation wavelength of 488 nm and emission wavelength of 530 nm with a bandwidth of 9 nm and 20 nm, respectively, and with a manual gain of 100. For mCherry fluorescence, an excitation wavelength of 550 nm and emission wavelength of 610 nm was used with a bandwidth of 9 and 20 nm, respectively, and a manual gain of 100. Fluorescence measurements on the microplate were normalized by optical density at 600 nm.

### Flow Cytometry and Cell Sorting

Overnight cultures were diluted 40-fold in 1× PBS for analysis with BD Accuri ™ C6 Plus flow cytometer (BD Biosciences) using excitation and emission wavelengths of 488 and 530 nm, respectively, to analyze sfGFP. For BD FACSAria™ III cell sorter (BD Biosciences) analysis and sorting, the cell samples were diluted ∼30-fold in 1× PBS, using standard settings for GFP (Excitation of 488 nm and Emission 530/30) or mCherry fluorescence (561 nm and 610/20). Parameters used for BD FACSAria™ are: FSC (Forward scattering) 1000, threshold SSC 200. Post FACS rounds, the cells were grown for ∼20 hr to saturation and then aliquoted and stored as glycerol stocks in a −80°C freezer.

### Depletion of Intracellular Chorismate Pool By UbiC

To evaluate the intracellular pool of chorismate, a pyruvate-lyase enzyme that converts chorismate into pyruvate and 4HB was inserted into the chorismate biosensor plasmid. Two versions of UbiC (wildtype and mutant UbiC-C22) were used for cloning into the chorismate sensor plasmid pJV5E.2. The latter is a double mutant (E31Q/M34V) of the *E. coli* UbiC and shows improved turnover in *P. putida* strain (Jha et al., 2019). *C. glutamicum* cells containing chorismate biosensor without UbiC expression and cells expressing UbiC_wt or UbiC_C22 were induced with 0, 0.1, 1, 3, and 10 mM of quinate when OD_600_ reached 0.6. Incubation at 30 °C was performed for 24 hr and 48 hr. The mCherry fluorescence was measured with BD FACSAria™ III cell sorter (BD Bioscience) and Infinite M200 Tecan plate reader.

### *qsuB* Gene Deletion in *C. Glutamicum*

*Corynebacterium glutamicum* ATCC 13032 (Δ*catB*) was transformed with the integrative plasmid pRH80, which uses the pK18sB backbone (Genbank: MH166772) (Jayakody et al., 2018) and 750 bp flanking regions of the *qsuB* gene. Gene deletions were performed by homologous recombination facilitated by Kanamycin (Km) selection and sucrose counterselection as previously described. Briefly, electroporated cells were plated onto plates containing Difco^TM^ Brain Heart Infusion (BHI) Agar (Becton Dickinson #241 830) supplemented with 25 μg/mL Kan. Single colonies were restreaked onto a second BHI/Km plate, then individual colonies from each re-streak were streaked onto BHI plates supplemented with 20% sucrose. Individual colonies were re-streaked on a second BHI +20% sucrose plate, then Km sensitivity of colonies was confirmed using a BHI and BHI/Km plate. Colony PCR was performed on Km sensitive colonies to confirm the deletion of *qsuB* from the *C. glutamicum* genome. The primers and plasmid sequences are described in Supplementary Table 1.

### *qsuD* Gene Deletion in *C. Glutamicum*

*Corynebacterium glutamicum* ATCC 13032 (WT) was transformed with the integrative plasmid pRH122 which contains a pK18sB backbone (Genbank: MH166772) (Jayakody et al., 2018) and with flanking regions of 793 bp (downstream) and 765 bp (upstream) near *qsuD* sequence. The *qsuD* gene deletion method was performed the same way as is described before for *qsuB* deletion gene (this work). The primers for deletion confirmation are described in Supplementary Table 1 (oBH049 and oBH050).

### Microscopy for *C. Glutamicum*

For microscopy assays, a frozen cell stocks of JV7 and JV13 were each inoculated into BHIS medium with antibiotics and cells were grown overnight in a shaking incubator at 30°C and 200 rpm, respectively. The next day, the cells were diluted (1:50) with fresh BHIS medium. When cells reached an OD_600_ of 0.6, they were transferred to a 96 deep well plate and quinate and/or benzoate were added to a final concentration of 3 mM. After 20 hr of incubation, 100 μL of cells were washed twice with PBS and fixed with 4% of paraformaldehyde for 15 min at room temperature. The cells were then washed twice with PBS and were stored at 4°C before mounting on a microscope slide using Prolong Glass Antifade Mountant (ThermoFisher Scientific) and cured for 2 days at room temperature as per manufacturer's instructions. Microscope imaging was performed on an Olympus IX83 with a UPLFL OPH 100 × 1.30 NA oil objective lens. The U-FBNA and U-FGNA mirror cubes were used for 494/518 nm and 555/580 nm excitation and emission wavelengths, respectively. Five images per sample were collected with multiple cells in the field of view. Images are exported as 16-bit grayscale TIFF files using Olympus cellSens Dimensions software and analyzed using Adobe Photoshop and a custom python script that can be found at the following GitHub repository: https://github.com/dmorales003/average_cell_intensity/tree/main/average_cell_intensity.

Due to the clumping of bacterial cells, a binary mask image was drawn by hand using Adobe Photoshop to identify the cell boundaries on the phase contrast image. An example is provided in Supplementary Fig. 1. The custom python script was then used to detect contours using the OpenCV package (Bradski, 2000) to segment and determine the mean pixel intensity of each cell using the Numpy package (Harris et al., 2020). A log transformation of the ratio of green to red fluorescence intensities was performed and plotted using the Pandas and Matplotlib library packages (Hunter, 2007; McKinney, 2010). Comparison of means was performed using the *t*-test function in *R* for a two-tailed *t*-test at a 95% confidence interval.

## Results

### *cis,cis*-Muconic Acid Biosensor in *C. Glutamicum* 13 032

**Fig. 2.**
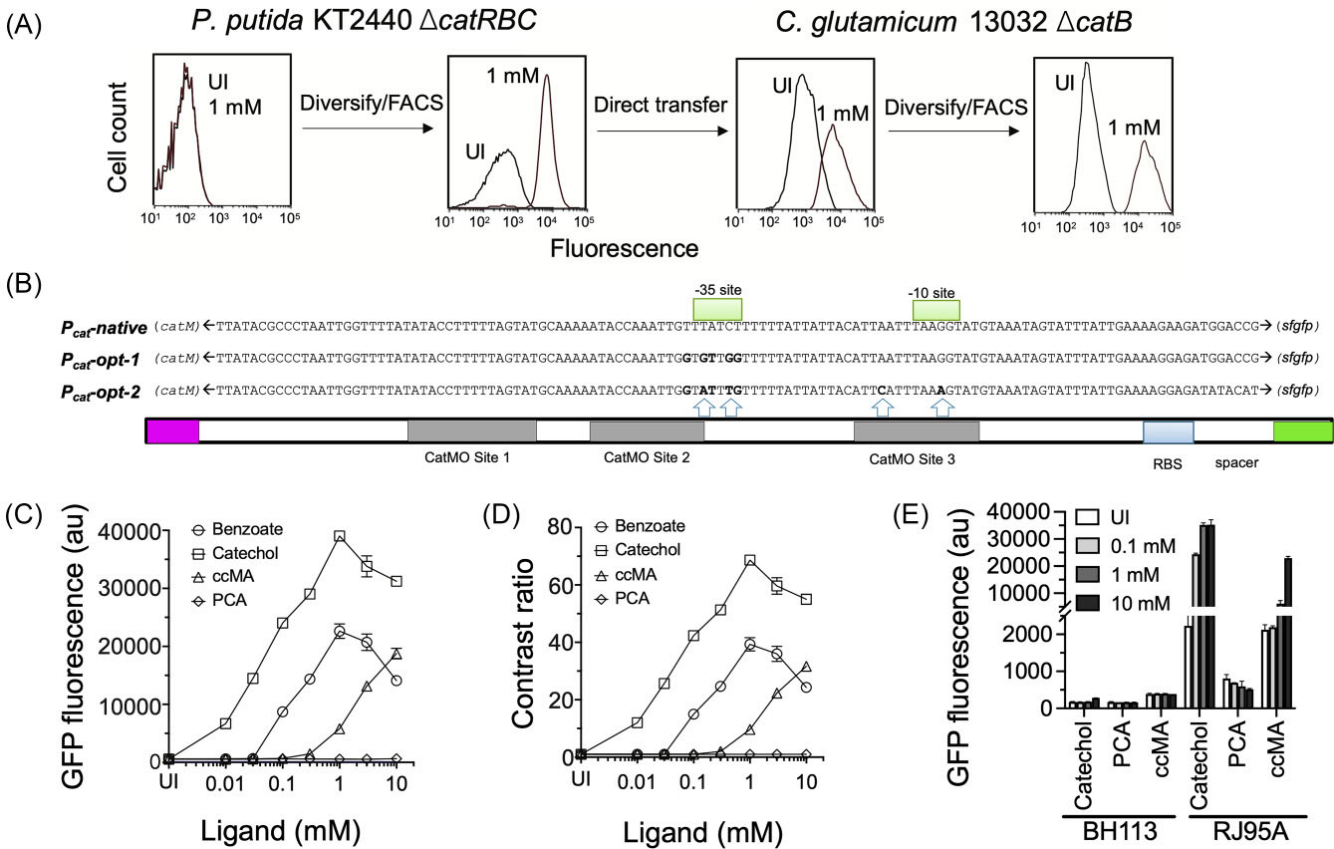
Development of a ccMA biosensor in *C. glutamicum*. (A) Whole cell fluorescence response with either no benzoate (UI) or 1 mM benzoate (a ccMA precursor) measured using a flow cytometer. Transfer of the *P. putida* optimized *P_cat_* promoter (*P_cat_*-opt-1) resulted in reduced response and high background in *C. glutamicum.* Using the same promoter library in *C. glutamicum*, another sequence (*P_cat_*-opt-2) was isolated that showed high dynamic range in *C. glutamicum.* (B) Mutations in *P_cat_*-opt-1 and *P_cat_*-opt-2 are shown in bold. The *P_cat_*-opt-2 promoter in *C. glutamicum* differed from the native promoter by seven base changes (marked in bold) and from the *P. putida* optimized promoter, *P_cat_*-opt-1, by four base changes (marked with arrows). (C) Raw sfGFP fluorescence of the ccMA biosensor in *C. glutamicum* in response to ccMA or ccMA precursors (benzoate or catechol). Protocatechuate (PCA) was used as a negative control, as *C. glutamicum* lacks the ability to convert PCA to ccMA. (D) Contrast ratio or dynamic range calculated as a fold change in fluorescence signal over uninduced (UI). (E) Comparison of ccMA biosensor response to ccMA or ccMA precursors (PCA and catechol). The biosensor in strain BH113 uses the native CatM-controlled promoter from *A. baylyi* ADP1, while the biosensor in strain RJ95A uses an optimized promoter *P_cat_*-opt-2. The data in (C), (D), and E represent mean values with standard deviation shown as error bars from three biological replicates. Error bars smaller than the symbol size are not displayed on the plot. Green fluorescent protein fluorescence signal represents mean fluorescence response of 100 000 individual cells measured using flow cytometer.

To develop a TF based ccMA biosensor in *C. glutamicum*, we first transferred the sensor-reporter cassette consisting of *catM* and *sfgfp* from the *P. putida* KT2440 based ccMA biosensor (pCatM_C2) (Bentley et al., 2020) to the *C. glutamicum* relevant vector pLFC007 consisting of a pBL1 origin of replication (Eikmanns et al., 1991). The genes *catM* and *sfgfp* encode for the CatM TF (Ezezika et al., 2006) and sfGFP (Pédelacq et al., 2006), respectively. The biosensor (plasmid construct pCg_CatM_C2, Supplementary Fig. 2, Table 1), when tested in the ccMA accumulating strain RH189 (*C. glutamicum* 13 032 Δ*catB*) (Becker, Kuhl, et al., 2018), showed a clear dose-response relationship with the ccMA precursor benzoate (Fig. S3). Benzoate is readily converted to ccMA in the presence of a functional β-ketoadipate pathway, while there was concern, based on previous work, that direct addition of ccMA could suffer from poor transport in the cell needing a ccMA-specific importer such as MucK (Shin et al., 2022). In the case of the ccMA biosensor in *P. putiida* (Bentley et al., 2020), we showed that benzoate could be converted to ccMA and trigger a response from the biosensor, and several subsequent knockout experiments demonstrated the biosensor's specificity for ccMA, that is, benzoate and other ccMA precursors could only activate the biosensor when their conversion routes to ccMA were intact. However, although there was a dose-response, the *P. putida* biosensor transferred to *C. glutamicum* also exhibited high background fluorescence and reduced contrast ratio [fold change in fluorescence over the uninduced condition (UI)] compared to when tested in *P. putida* (Fig. 2a). This result prompted us to revisit the *P_cat_* promoter library constructed previously for *P. putida* (Bentley et al., 2020). Transformation of *C. glutamicum* with this promoter library (theoretical diversity >65 000) resulted in only partial library coverage (∼35 000 or 54% of total diversity). Considering that we previously observed multiple high performing P*_cat_* variants when screening for the ccMA biosensor in *P. putida*, we hypothesized that even an under-represented library would fetch an improved ccMA biosensor for *C. glutamicum.* Therefore, we pursued the growth, induction and FACS of the transformed library in *C. glutamicum* and performed three rounds of positive selection (selecting for high fluorescence in the presence of 1 mM benzoate) and a single round of negative selection targeting low background fluorescence in an uninduced population. This resulted in multiple promising clones that exhibited contrast ratios >20 for the ccMA precursors, benzoate, or catechol, added at 1 mM. The promoter sequence in clone RJ95A (harboring biosensor plasmid pRJ2010 and promoter sequence *P_cat_*-opt-2) had four base changes compared with the optimized promoter for *P. putida, P_cat_*-opt-1 (Fig. 2b). This clone produced a >65-fold contrast ratio upon the addition of 1 mM catechol and produced a detectable signal at catechol concentrations of <10 μM. Upon the addition of 1 mM benzoate, the clone exhibited >40-fold contrast ratio, and the lowest detected benzoate concentration was >30 μM (Fig. 2c–d). At concentrations higher than 1 mM, the fluorescence response decreased, possibly due to toxicity effects from the aromatic precursors, that was evident by the lower cell density in those cultures. A negative control precursor, protocatechuate (PCA), which does not have a metabolic route for conversion to ccMA in *C. glutamicum* (due to lack of a PCA decarboxylase gene, *aroY*) (Lee et al., 2018), failed to show any significant fluorescence response even at a concentration of 10 mM (Fig. 2c). Overall, *P_cat_*-opt-2 contained seven mutations in the −35/−10 and CatMO region when compared to the *P_cat_*_-_native promoter (Fig. 2b). These changes to the promoter, in combination with a strong RBS site that was introduced in *P_cat_*-opt-2, resulted in a higher sensitivity and dynamic range, compared to the *P_cat_*-native promoter (Fig. 2e).

**Table 1.** Plasmids and strains used in this work.

Plasmid	Description	References/Sources
pLFC007	*Corynebacterium glutamicum*/*E. coli* shuttle vector with pBL1/ColE1 origin of replications; Cm^R^, *araE, araC* and *eGFP* gene under control of the *P_BAD_* promoter	Taek Soon Lee Lab, Joint Bioenergy Institute
pCatM_C2	*cis,cis*-muconic acid biosensor construct for *P. putida* KT2440 consisting of the *P_cat_*-opt-1 promoter and *catM* sensor and *sfgfp* fluorescent reporter	(Bentley et al., 2020)
pCg_CatM_C2	*cis,cis*-muconic acid sensor-reporter cassette from pCatM_C2 transferred to pLFC007 backbone	This work
pCg_CatM_promo_Lib1	*cis,cis*-muconic acid sensor-reporter cassette with diversified promoter region and canonical RBS	Adapted from (Bentley et al., 2020)
pRJ2010	*cis,cis*-muconic acid biosensor construct with *P_cat_*-opt-2 promoter, derived from screening the *P_cat_* promoter library	This work
pALC412	Temperature-sensitive replicating vector; contains ApmR for selection in *C. glutamicum*, and a mutation in RepA, proline (CCT) to serine (TCT) that allows for maintenance in *C. glutamicum* below 30˚C and temperature-curing above 37˚C as described in Nakamura et al. (2006). Also contains pMB1 and bla genes for maintenance in *E. coli*	(Nakamura et al., 2006) This work
pJV1	Chorismate biosensor plasmid carrying *mCherry* reporter gene under control of *P_qsu_*, a native promoter in *C. glutamicum*	This work
pJV4	Temperature sensitive ccMA biosensor for *C. glutamicum*. ccMA sensor-reporter cassette from pRJ2010 inserted into pALC412 plasmid, containing ApmR and bla genes for maintenance in *E. coli*.	This work
pJV5	Diversified promoter *P_qsu_* for chorismate biosensor in *C. glutamicum*	This work
pJV5E.2	An optimized promoter selected from pJV5 for chorismate sensing in *C. glutamicum*	This work
pJV8	Dual biosensor for sensing ccMA and chorismate with optimized *P_cat_* and *P_qsu_* promoters activating sfGFP and mCherry expression, respectively	This work
pJV9	*ubiC-wt* gene under *P_dapA_* promoter inserted into pJV5E.2 for validation of the chorismate biosensor	This work
pJV10	Activity enhanced mutant *ubiC_C22* gene under *P_dapA_* promoter inserted into pJV5E.2 for validation of the chorismate biosensor	This work
**Strains**	**Genotype**	**References/Sources**
*C. glutamicum*	*Corynebacterium glutamicum* strain ATCC 13 032	ATCC 13 032
RH189	*Corynebacterium glutamicum* Δ*catB*	This work
RJ89A	*Corynebacterium glutamicum* Δ*catB* harboring *P_cat_* promoter library pCg_CatM_promo_Lib1	This work
RJ95A	*Corynebacterium glutamicum* Δ*catB* harboring pRJ2010	This work
JV1	*Corynebacterium glutamicum* harboring pJV1	This work
JV5E.2	*Corynebacterium glutamicum* harboring pJV5E.2	This work
JV7	*Corynebacterium glutamicum* Δ*catB* harboring pJV8	This work
JV13	*Corynebacterium glutamicum* Δ*catB* Δ*qsuB* harboring pJV8	This work
BH113	*Corynebacterium glutamicum* Δ*catB* harboring ccMA biosensor construct with *P_cat_*-native sequence	This work
BH120	*Corynebacterium glutamicum* Δ*catB* Δ*qsuD* harboring pJV5E.2	This work

### ccMA Transport in *C. Glutamicum*

Having developed a functional ccMA biosensor in *C. glutamicum*, we tested the response directly from extracellular ccMA supplementation. Due to the presence of two carboxylic acid groups in ccMA, which are expected to be deprotonated at pH ∼7 (our experimental conditions), passive diffusion through the cell membrane will be energetically unfavorable, likely requiring a transporter to facilitate its import across the cell membrane. RJ95A showed a dose-dependent response to ccMA when added at concentration greater than 0.3 mM, with the maximum fold-change in fluorescence reaching >30 at 10 mM ccMA (Fig. 2c–d). In contrast, the *P. putida* optimized ccMA biosensor in *P. putida* responded negligibly to exogenous ccMA addition, at least up to 1 mM (Fig. S4) and approximately 1.7-fold at concentrations as high as 10 mM (Shin et al., 2022). Introduction of the muconate transporter MucK facilitated ccMA transport in *P. putida*, resulting in a very high biosensor response at 1 mM ccMA (Supplementary Fig. 4) (Shin et al., 2022). These data suggest that ccMA can be taken up from the medium by *C. glutamicum*; however, a bioinformatics search of the *C. glutamicum* genome (NCBI reference sequence NC_003450.3) revealed no annotated ccMA transporters.

### Development of a Chorismate Biosensor in *C. Glutamicum*

**Fig. 3.**
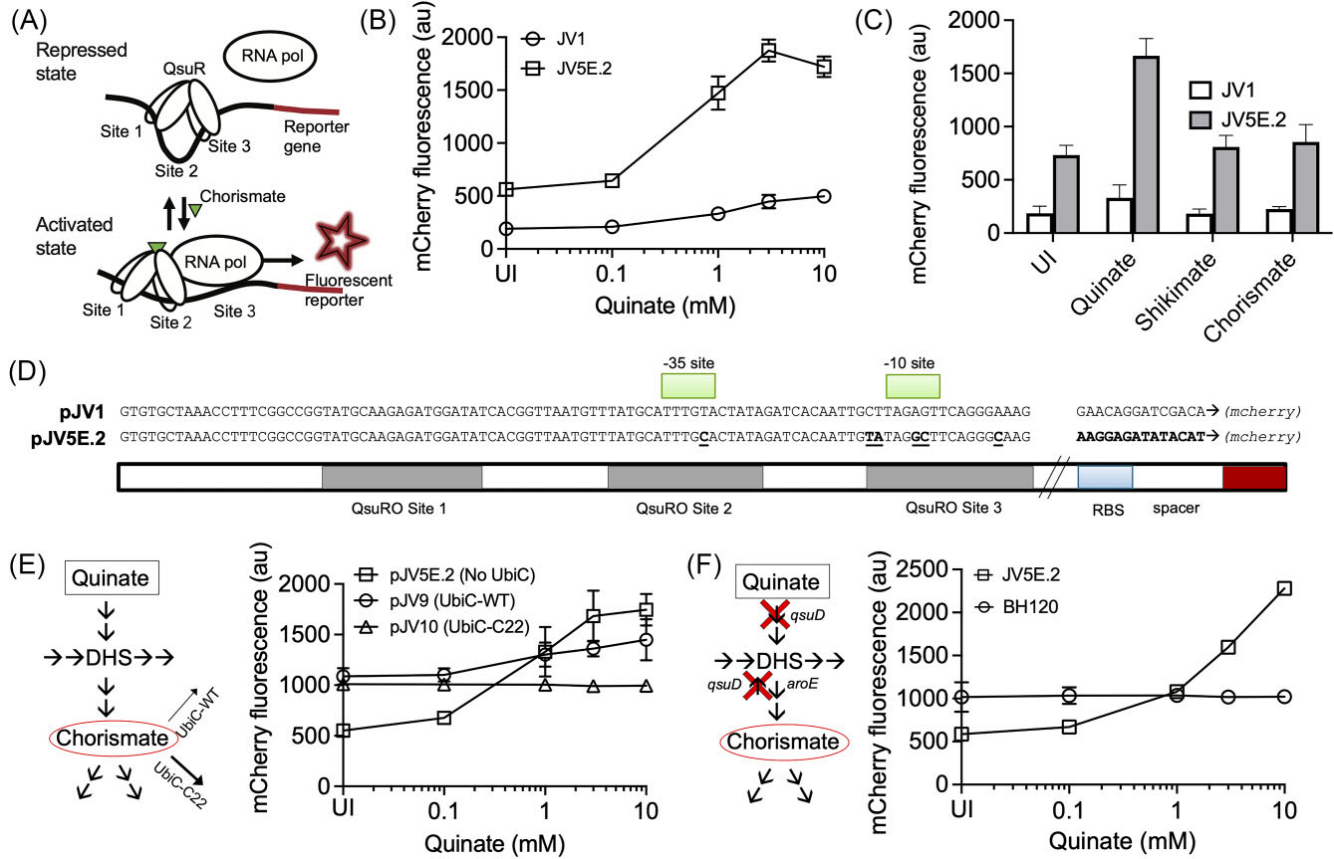
Development of a chorismate biosensor in *C. glutamicum* ATCC 13032. (A) Illustration of QsuR-mediated activation of transcription in the presence of chorismate and its utility in biosensor development when used in conjunction with *mCherry* reporter gene. Sites 1, 2, and 3 represent three QsuR operator (QsuRO) sites. (B) Dose-response of native (pJV1) and optimized (pJV5E.2) biosensor variants evaluated in *C. glutamicum* using quinate as a chorismate precursor (UI, uninduced). (C) The *C. glutamicum* biosensor response to chorismate and the chorismate precursors, shikimate and quinate (UI, uninduced). (D) Sequence comparison of operator and promoter regions of the native and optimized chorismate biosensors. The region between QsuRO site 3 and the RBS (ribosome binding site) were unchanged (not shown). (E) Investigation of QsuR-based biosensor specificity by UbiC mediated depletion of the intracellular chorismate pool. Native *E. coli* UbiC is represented as UbiC-WT and a double mutant of UbiC with alleviated product inhibition is referred to as UbiC-C22 that introduce weak and strong depletion of chorismate, respectively. (F) QsuR-based biosensor in the *qsuD* knockout strain (BH120) that cannot covert quinate to 3-dehydroquinate and hence entry into the shikimate pathway. The data represent mean values with standard deviation shown as error bars from three biological replicates. Error bars smaller than the symbol size are not displayed on the plot. mCherry fluorescence signal represents mean fluorescence response of 100 000 individual cells measured using flow cytometer.

QsuR, a LysR family transcription regulator is annotated as a chorismate-dependent regulator of genes involved in shikimate and quinate utilization in *C. glutamicum*, and an activator of *qsuA* gene expression via the *P_qsu_* promoter (Kubota et al., 2014). To develop a chorismate biosensor, the vector backbone was acquired from pRJ2010, and the *catM-P_cat_*-opt-2-*sfgfp* gene cassette was replaced with *P_qsu_*-*mCherry* nucleotide sequence, where *mCherry* encodes for the monomeric cherry fluorescent protein (mCherry) (Shaner et al., 2004). The *P_qsu_* promoter is an intergenic region between the sequences of the antiparallel genes CGL_RS02135 (*qsuR*) and CGL_RS02140 (*qsuA*) in the genome of *C. glutamicum* (NC_003450.3) (Supplementary Fig. 5a). The proposed mechanism for the resulting biosensor is shown in Fig. 3a and assumes that QsuR functions similarly to most LysR family transcription regulators. Since QsuR is natively expressed in the *C. glutamicum* cells, the gene for this TF did not need to be inserted in the biosensor plasmid (Supplementary Fig. 5b). *Corynebacterium glutamicum* cells harboring the chorismate biosensor construct (pJV1) were grown in the presence of exogenous quinate and shikimate to test the biosensor. Exogenous chorismate was not directly used to induce the biosensor, because chorismate is unstable and likely does not import easily through the cell membrane (Gibson & Pittard, 1968). In *C. glutamicum*, attempts to induce QsuR-regulated genes with extracellular chorismate failed, but both quinate and shikimate, which are metabolized to chorismate via the shikimate pathway, have been previously shown (in a related strain *C. glutamicum* strain R) to play role in the induction of QsuR-regulated genes, even though the QsuR interacts with chorismate specifically and not with quinate or shikimate (Kubota et al., 2014). Furthermore, in that strain deletion of *aroC* gene (encoding chorismate synthase) showed disappearance of quinate/shikimate dependent induction of QsuR-regulated genes (Kubota et al., 2014). Attempts to elicit a fluorescent response to exogenously added shikimate failed in *C. glutamicum* ATCC 13032 strain (possibly due to a permeability barrier and absence of any shikimate transporter as verified from the genome sequence NC_003450.3). However, quinate addition resulted in a dose-dependent fluorescent response (between 0.1 mM and 3 mM quinate supplementation), with a contrast ratio of less than twofold (Fig. 3b).

To further enhance the sensitivity and the dynamic range of the QsuR based biosensor, we partially or completely randomized specific regions in the *P_qsu_* promoter to create a library of theoretical diversity of ∼260 000 (Supplementary Fig. 5c). The mutations in the QsuR operator and −35/−10 promoter regions were targeted to perturb the switch that QsuR undergoes for chorismate-mediated activation of transcription (Fig. 3a). The RBS and the spacer upstream of *mCherry* was switched to the sequence consistent with the ccMA biosensor (RBS: AAGGAGA, spacer: tatacat, Fig. 2b) to increase the amplitude of the reporter signal. After a few rounds of FACS, one of the isolated variants (pJV5E.2), showed increased sensitivity and dynamic range in response to the quinate concentrations (Fig. 3b). The signal in the absence of quinate (background signal) increased by approximately threefold compared with the native promoter response, however a contrast ratio of fourfold was also achievable in the optimized biosensor (Fig. 3b). The biosensor was not activated, however, by induction with shikimate or chorismate (Fig. 3c). Sequencing confirmed that the mutations were in the targeted regions of our focused library and consisted of combinations of mutations in the operator and the promoter regions (Fig. 3d).

### Validation of QsuR-based Biosensor As a Chorismate Biosensor

Since the dose-response of the QsuR-based biosensor was performed using quinate, we further investigated if the biosensor response was due to quinate, chorismate, or other metabolites inside the cell (Fig. 1). While the possibility of quinate or downstream metabolites activating the biosensor has been previously investigated (Kubota et al., 2014), we targeted perturbing the chorismate pool for further evaluation. Introduction of a heterologous chorismate pyruvate-lyase (UbiC) would convert chorismate to pyruvate and 4-hydroxybenzoate, depleting the intracellular chorismate pool, and potentially affecting QsuR-based biosensor response. In our previous work, we engineered a variant of *E. coli* UbiC (UbiC-C22) with substantially alleviated product inhibition and showed improved UbiC bioconversion in *P. putida* (Jha et al., 2019). The genetic sequences of wildtype *ubiC* (*ubiC-wt)* and *ubiC-C22* were introduced in the pJV5E.2 plasmid under a weak *P_dapA_* promoter (Vašicová et al., 1999). Attempts to insert plasmids with *ubiC* genes expressed with a strong promoter, such as *P_tac_*, into *C. glutamicum* were not successful. The two new variants of the chorismate biosensor plasmid that also expressed UbiC-WT or UbiC-C22 from the same plasmid, pJV9 and pJV10, respectively (Fig. S6), were compared with the original QsuR-based biosensor-only construct, pJV5E.2. Expression of UbiC from the biosensor plasmid resulted in reduction or loss of quinate-induced fluorescence (Fig. 3e). The activity difference between UbiC-WT and UbiC-C22 correlated with the biosensor response; the UbiC-C22 variant suppressed the biosensor response more strongly than UbiC-WT. A possible argument could be made that the UbiC mediated loss of dose-response is an effect of quinate depletion achieved by stronger pull from UbiC, but considering quinate is much upstream in the shikimate pathway, UbiC mediated chorismate depletion is expected only minimally perturb the intracellular pool of quinate, which was used at a concentration as high as 10 mM in the dose-response experiment.

To further probe this, we knocked out the *qsuD* gene (non-essential for survival), which encodes a quinate/shikimate dehydrogenase involved in two reactions that link quinate carbon to the shikimate pathway. QsuD catalyzes the oxidation of quinate to 3-dehydroquinate (3DHQ) and the oxidation of shikimate to 3-dehydroshikimate (3DHS) (Fig. 1) (Kubota et al., 2013; Teramoto et al., 2009). The biosensor strain BH120 (*qsuD* knockout) did not exhibit a quinate-dependent fluorescence (Fig. 3f), further validating that quinate is not an effector molecule for QsuR. In BH120, we also observed a marginal increase in the baseline response from the QsuR biosensor, which may be attributed to higher carbon flux towards chorismate due to reduced shikimate to 3DHS bioconversion (Fig. 1) (Kubota et al., 2013). Overall, the results suggest that the QsuR-based biosensor responds primarily to chorismate in *C. glutamicum.*

### Sensing Two Metabolites Simultaneously With the ccMA and Chorismate Biosensors in *C. Glutamicum*

*cis,cis*-muconic acid and chorismate are key metabolites from the shikimate and β-ketoadipate pathways. The two pathways can be connected by bioconversion of dehydroshikimate (Draths & Frost, 1994; Bentley et al., 2020) or chorismate (Jha et al., 2019) using a few enzymes (Fig. 1). In wild type *C. glutamicum*, QsuB connects the shikimate and the β-ketoadipate pathways, but the absence of a native PCA decarboxylase (AroY) prevents carbon flux from the shikimate pathway to ccMA. However, in other microbial hosts lacking a native PCA decarboxylase (e.g., *P. putida*) carbon flux has been successfully diverted to ccMA from the shikimate pathway by introduction of a functional AroY, providing a promising synthetic pathway for production of ccMA from simple sugars (Bentley et al., 2020; Ling et al., 2022). Thus, we were motivated to establish dual sensing of chorismate and ccMA in *C. glutamicum.*

**Fig. 4.**
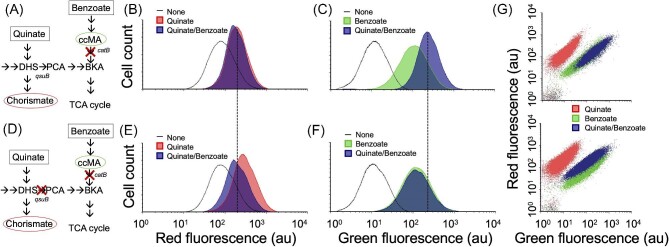
ccMA/chorismate dual biosensing in *C. glutamicum*. (A) Illustration of metabolic pathways and expected fluorescence response in *C. glutamicum* variant JV7. (B) Fluorescence response of JV7 to quinate and quinate/benzoate as measured in the red channel (ex/em 561/610). (C) Fluorescence response of JV7 to benzoate and quinate/benzoate as measured in the green channel (ex/em 488/530). (D) Illustration of metabolic pathways and expected fluorescence response in *C. glutamicum* variant JV13. (E) Red fluorescence response of JV13 to quinate and quinate/benzoate. (F) Green fluorescence response of JV13 to benzoate and quinate/benzoate. (G) 2-D scatter plot of JV7 (top) and JV13 (bottom) showing fluorescence in red and green channels. The data were collected using BD FACSAria III flow cytometer. Ligand concentrations were fixed at 3 mM of quinate or benzoate or 3 mM of each for the mixed supplement. The blue color/darker shade overlay in each histogram and in the scatter plot is a representation of the cell induction with both quinate/benzoate. The quinate response in the green channel and benzoate response in the red channel are not shown for clarity but were indistinguishable from the no supplement control.

The *P_qsu_*-*mCherry* cassette with a dual transcription terminator (Supplementary Fig. 5b) was transferred to the ccMA biosensor construct pRJ2010 to create a ccMA/chorismate dual biosensor plasmid, pJV8. The dual biosensor plasmid (Supplementary Fig. 7) was tested in the *C. glutamicum* Δ*catB* strain (Fig. 4a). The strain, dubbed JV7 was titrated with quinate (chorismate precursor) or benzoate (ccMA precursor) and showed a dose-dependent response to both substrates as detected by red (mCherry) or green (sfGFP) fluorescence signal intensity, respectively (Fig. 4b–c). However, the dynamic range of both the ccMA and chorismate biosensors showed reduction, which may be attributed to increased metabolic burden due to the energy demand of two functional biosensors. Unexpectedly, the ccMA biosensor in this strain exhibited an enhanced response to benzoate when concurrently exposed to quinate (Fig. 4c, Supplementary Fig. 8), an effect that was observed at a concentration as low as 0.1 mM quinate (Supplementary Fig. 9). In *C. glutamicum*, the shikimate pathway and ccMA production branch of β-ketoadipate pathway are not connected (absence of PCA decarboxylase, AroY) and should not crosstalk with each other, so the high ccMA biosensor response in the presence of quinate was indeed surprising and hence, needed further probing to gain understanding.

To verify if the quinate-dependent increase in ccMA biosensor response could be attributed to an increase in carbon flux in the shikimate or β-ketoadipate pathway, the dual biosensor pJV8 was tested in a strain where the gene (*qsuB*) that connects the shikimate pathway with β-ketoadipate pathway was deleted (Figs. 1 and 4d). In response to quinate, the new strain JV13 showed an increase in red fluorescence intensity compared to JV7, which could be attributed to an increased carbon flux in shikimate pathway that leads to an increase in the chorismate pool (compare Fig. 4b and e). In JV13, the green fluorescence response from ccMA biosensor with benzoate and quinate/benzoate were indistinguishable (compare Fig. 4c and f). To further visualize the data, the red and green fluorescence intensities were plotted together for the two strains JV7 and JV13 with different inducer feedings. The scatter plot for JV7 (Fig. 4g, top) shows that the simultaneous feeding of quinate/benzoate affected the signal in the green channel (ccMA biosensor response) higher than the benzoate alone. In JV13, the observed response is an additive effect of quinate increasing the chorismate biosensor response and benzoate increasing the ccMA biosensor response, as the two pathways cannot crosstalk anymore due to deletion of the *qsuB* gene (Fig. 4g, bottom). To test if the quinate-dependent increase in the ccMA biosensor was specific to the dual biosensor plasmid, we tested the individual ccMA biosensor (strain RJ95A) under a similar growth and induction condition and saw a shift in the histogram towards an increase in green fluorescence when quinate/benzoate were added together (Supplementary Fig. 10).

### Evaluation of *C. glutamicum* Biosensor Response Using Microscopy

**Fig. 5.**
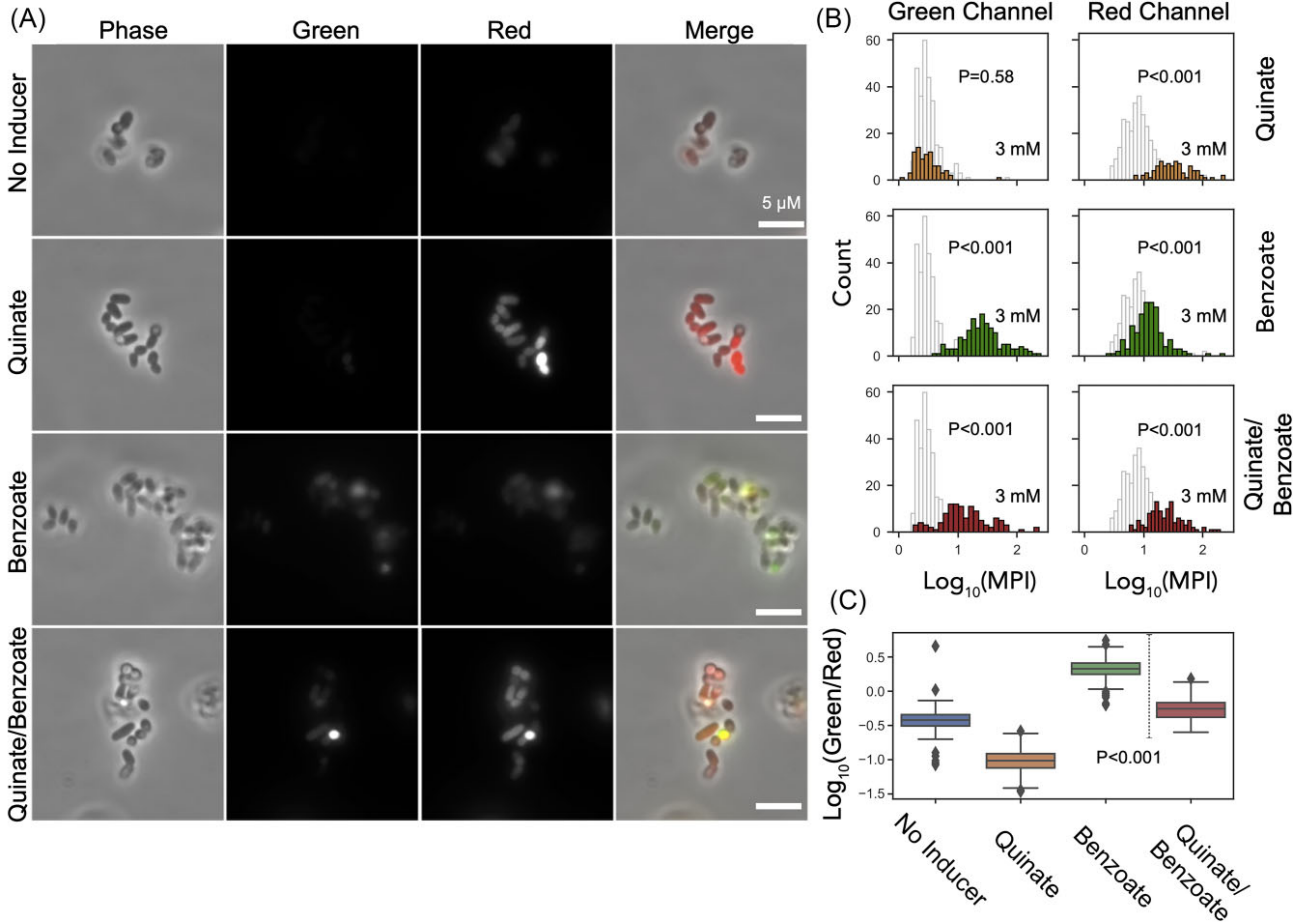
Visualizing fluorescence response from the dual biosensor in *C. glutamicum* using microscopy. JV13 cells (*C. glutamicum* Δ*catB* Δ*qsuB*) were grown under chorismate and/or ccMA precursors, such as quinate and benzoate respectively. (A) Phase and fluorescence imaging of the dual biosensors in *C. glutamicum* performed by microscopy. Spherical and cylindrical populations of cells are observed. Segmentation masks were generated from phase images to perform single-cell analysis. Scale bar is 5 µm. (B) Log transformed mean pixel intensities (MPI) of individual cells are plotted for each treatment (filled bars) and compared against the non-induced cells (unfilled) for both green and red fluorescence. Significant differences in the means were observed for fluorescence profiles. (C) Log transformation of the ratio between green and red fluorescence in single cells for each precursor addition. A significant shift was observed in the average ratio between benzoate treated and co-treated (quinate and benzoate) cells, indicating a clear distinction between the two populations. Comparison of mean values were performed by a two-tailed Student's *t*-test at a 95% confidence interval.

We further probed single-cell morphology and fluorescence intensity of the *C. glutamicum* reporter strains using fluorescence microscopy. The *C. glutamicum* cells showed noticeable clumping of two distinct morphologies (spherical and rod-shaped) (Fig. 5a, Phase contrast). There was no clear evidence that one morphology was predominant over the other when carbon flux in the shikimate pathway was increased by adding the precursor quinate, leading to an activation of the chorismate biosensor. Similarly, there was no observed difference in the cell morphology when the production of a non-essential metabolite such as ccMA was increased, which led to an activation of the ccMA biosensor (Fig. 5, Supplementary Fig. 11). Fluorescence microscopy showed a baseline red fluorescence within the cells, while the green fluorescence had little to no baseline fluorescence in the absence of exogenous inducers (e.g., quinate and/or benzoate), consistent with the observation of a relatively high red fluorescence baseline in the flow cytometry analyses. Reporter fluorescence was commensurate with the activation of the associated cell biosensor, where the fluorescence intensity was strongly enhanced in the chorismate biosensor strain with the addition of 3 mM quinate and in the ccMA biosensor strain upon the addition of 3 mM benzoate (Fig. 5a, green and red panels). Additionally, merging the two fluorescence channels showed colocalizing green and red fluorescence (Yellow) when both treatments were performed simultaneously (Fig. 5a, Merge panel). Comparison of the distribution of the mean-pixel intensity (MPI) for individual cells showed that reporter fluorescence intensities were significantly different (*P* < 0.001 for a 95% confidence interval) from non-induced cells (Fig. 5b). The reporters also showed increased incidence of cells with red fluorescence when treated with benzoate, suggesting potential cross-activation. To dispel the ambiguity regarding the cellular response to the precursors, ratio analysis of the green and red MPI of single cells showed dominant red or green fluorescence for the cells treated with quinate or benzoate respectively as represented by the ratios less than or greater than 0 after log transformation (Fig. 5c). These ratios show a significant difference (*P* < 0.001) in cells treated with benzoate compared to the cells treated with both inducers, which was comprised of predominantly red expressing cells (*P* < 0.001, compared to *mu* = 1), indicating that these populations can be separated. Cells of strain JV13 (JV7 Δ*qsuB*) characteristically had greater red fluorescence intensity than those of JV7 when treated with quinate (*P* < 0.001, one-tailed *t*-test at 99.5% confidence interval), indicating a greater signal to noise for the 3 mM quinate response. Additionally, JV13 and JV7 presented similar green fluorescence intensities when treated with benzoate (*P* = 0.941, two-tailed *t*-test at 99.5% confidence interval), suggesting similar responsiveness to the product, ccMA for both biosensors (Fig. S12).

### Temperature-Sensitive Biosensor Plasmid for Rapid Curing

**Fig. 6.**
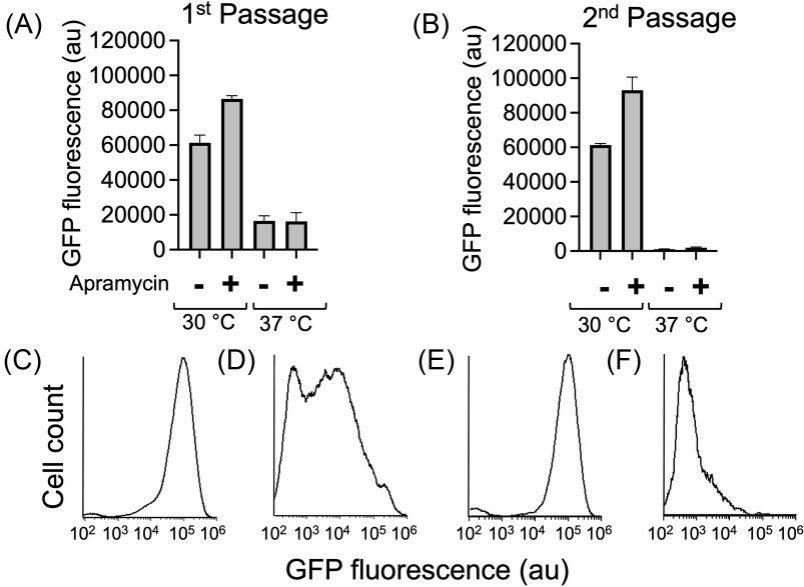
Development of a temperature sensitive (ts) ccMA biosensor in *C. glutamicum* for rapid curing. (A) First round of growth and induction of *C. glutamicum* Δ*catB* strain harboring ts ccMA biosensor, pJV4. The biosensor consists of an apramycin resistance gene. Induction was performed using 1 mM catechol and the cells were grown at 30°C or 37°C in the presence or absence of apramycin. (B) Second round of growth and induction while maintaining the same conditions as the first round. (C) *Corynebacterium glutamicum* cells after first passage grown at 30°C with apramycin; (D) after first passage grown at 37°C, no apramycin; (E) after second passage grown at 30°C with apramycin; (F) after second passage grown at 37°C, no apramycin. The (C–F) histograms were generated by BD Accuri C6 flow cytometer.

While biosensor tools provide an increase in the throughput for screening strain phenotypes, the ability to cure the strain of the biosensor becomes a necessary step for downstream microbial engineering efforts so that the strain's performance can be evaluated without the metabolic burden of the biosensor. To establish a protocol for curing strains of the biosensor plasmids, we ported the sensor-reporter cassette from the ccMA biosensor plasmid pRJ2010 to pALC412, a temperature-curable vector. pALC412 contains an apramycin resistance gene, a pBL1 replicon, and a RepA replicase with a proline to serine mutation that makes it temperature sensitive and allows for temperature-dependent replication in *C. glutamicum* (Nakamura et al., 2006). The resulting plasmid, pJV4 (Supplementary Fig. 13), was transformed into the *C. glutamicum* Δ*catB* strain, and the fluorescence response from the ccMA biosensor was measured upon the addition of catechol (a ccMA precursor). To assess curing of the vector, cells were exposed to catechol at both 30°C and 37°C in the presence and absence of apramycin. The fluorescence response from the ccMA biosensor was used to determine the relative level of plasmid maintenance in the strain and whether temperature curing was successful. At 30°C under apramycin selection, pJV4 was relatively stable in *C. glutamicum* (Fig. 6a and c), whereas at 37°C there was substantial decrease in plasmid maintenance as measured by the catechol induced fluorescence response of the population. Flow cytometry analysis showed heterogeneity in the population, with a mix of reduced (possibly due to decrease in copy number of plasmid) and no fluorescence (plasmid cured) cells (Fig. 6a and d). When the cells from above condition were regrown under the same condition, a homogeneous population was observed at 30°C with apramycin and strong GFP fluorescence was retained (Fig. 6b and e). At 37°C, the cell density was quite low both in the presence and absence of apramycin, but the cells were completely devoid of green fluorescence signal (Fig. 6b and f).

## Discussion

Transcription factors are promising scaffolds for establishing biosensors in an organism. Although their promoters are active for native function, they usually need to be optimized for sensitivity, linear detection range, and contrast ratio (dynamic range) to increase their utility for biotechnological applications. There are several key regions in a TF and promoter that can be mutated for gain-of-function in a biosensor. Mutations in the −35/−10 and/or operator sites have led to increases in sensitivity and dynamic range and made TFs usable in new host organisms (Jha et al., 2014, 2018; Bentley et al., 2020; Pardo et al., 2020). More specifically, because the mechanism of transcription activation and controlling features of various LTTRs are often reasonably conserved, they are good targets for semi-rational library design for biosensor development. An LTTR is a homo-tetramer that binds to at least two locations near the transcriptional initiation site (TI). The regulatory binding site typically positioned upstream from the TI binds the LTTR, which remains strongly anchored in either the apo form or when bound to the co-inducer. The other arm of the tetramer binds to an activation binding site with lower affinity. Conformational changes in the protein upon binding to the co-inducer shifts the binding position of the second arm marginally in some cases, or to a third LTTR-binding motif entirely in other cases. These shifts can alter DNA bending, expose important promoter regions or allow direct interaction between the LTTR and the RNA polymerase complex (Maddocks & Oyston, 2008). In the current study, the TFs belonged to the LTTR family and the knowledge of this class of TFs was applied for establishing biosensors in *C. glutamicum* ATCC 13032.

In the current work, we pursued semi-rational design of promoter libraries for the development of biosensors for ccMA and chorismate (Masuo et al., 2016). We used a heterologous LTTR, namely CatM from *A. baylyi* ADP1 as a ccMA biosensor, while the native QsuR, also belonging to the LTTR family, was used for engineering a chorismate biosensor. Libraries of size on the order of ∼10^5^ were built and screened using flow cytometry in both biosensor development workflows. While direct transfer of a biosensor cassette from one host to another can compromise its function, we were able to exploit a CatM regulated promoter library developed for *P. putida* to screen and isolate a promoter variant with much improved response in *C. glutamicum* in an expedited manner. The native *P_cat_* promoter from *A. baylyi* ADP1 failed to show any detectable dose-response with ccMA precursors, but seven mutations in the promoter and operator regions along with a strong RBS generated high sensitivity and dynamic range in response to ccMA (Fig. 2e). In the case of the chorismate biosensor, a new promoter variant with six mutations and a strong RBS helped increase the sensitivity and dynamic range in response to feeding of quinate (chorismate precursor).

Interestingly, and contrary to the functional ccMA biosensor in *P. putida*, we observed a response of this biosensor in *C. glutamicum* to the addition of exogenous ccMA to the growth medium. Although there are substantial differences in the membrane composition of *P. putida* (Gram-negative) and *C. glutamicum* (Gram-positive), it is still unlikely that any sufficient diffusion of ccMA across the membrane took place as it will carry a net −2 charge at the physiological pH. Thus, we hypothesized that the biosensor response was due to ccMA import facilitated by an active transporter. The fact that the ccMA biosensor in *C. glutamicum* showed response to extracellular ccMA at 10-fold to 100-fold higher concentrations than observed using the ccMA precursors benzoate and catechol (Fig. 2d), a wide range of transporters might be promiscuously operating to transport ccMA, albeit poorly. Our investigation was biased towards MFS transporter-mediated ccMA transport in *C. glutamicum* since an annotated ccMA transporter, MucK belongs to the same family. A survey into the genome of *C. glutamicum* ATCC 13032 using MucK from *A. baylyi* ADP1 (Sequence ID P94131.1) revealed the presence of a relevant MucK homolog with only ∼27% sequence identity (Sequence ID BAC00372.1, Supplementary Information SI 2.2) in this strain, a protein regarded as an MFS transporter. Close homologs of this MFS transporter identified in other *Corynebacterium* strains were also annotated as a hypothetical protein (Sequence ID BAF55884.1, Supplementary Information SI 2.4.1) or as a sugar phosphate permease (Sequence ID WP_047263143.1, Supplementary Information SI 2.4.2) We surmise that the transporters of other families could also achieve the same task, as suggested by the discovery of several C_4_ dicarboxylate transporters, such as DccT and DctA in *C. glutamicum* (Youn et al., 2008, 2009). Future work that further investigates the role of such genes in transport of dicarboxylic acids will provide insights and gene knockouts could be used to measure the effect on ccMA transport using the ccMA biosensor.

Shikimate is an intermediate metabolite in the shikimate pathway upstream of chorismate and can be used as a precursor to alter the intracellular chorismate pool. A shikimate biosensor was previously developed in *C. glutamicum* RES167 (derivative strain of *C. glutamicum* ATCC 13032) that showed a dose-dependent response to shikimate only when a heterologous gene *shiA* encoding for the shikimate transporter and natively present in *C. glutamicum* strain R (JCM18229) (Kubota et al., 2015), but not in *C. glutamicum* RES167, was included in the biosensor plasmid (Liu et al., 2018). In that study, cells grew poorly with shikimate as the sole carbon source, and the biosensor responded only weakly to exogenous shikimate until *shiA* was introduced on the biosensor plasmid, which improved cell growth and increased the fluorescence response. This may be why we did not observe a dose-dependent response to exogenously added shikimate. Like Liu et al. (Liu et al., 2018), we were unable to find a *shiA* homologue, nor any other suitable gene target that could transport shikimate in *C. glutamicum* ATCC 13032. We expected some cross reactivity in functions of transporter proteins for quinate and shikimate (due to similarity of the two molecules), specifically that the quinate transporter QsuA could act as a transporter for shikimate, but we failed to detect any increase in the chorismate biosensor response upon exogenous shikimate addition (Fig. 3c). By contrast, Kubota et al. observed activation of the QsuR-controlled operon by shikimate in a *C. glutamicum* strain R and but this be attributed to the presence of the *shiA* gene coding for a shikimate transporter in that particular strain.

Both biosensors have their limitations. As with most TF based fluorescence biosensors, they become unreliable below a certain sensitivity threshold or above a certain saturation threshold. In this study, the biosensors were induced with the external addition of precursor metabolites rather than directly with the ligands for the TFs for various transport and stability reasons. As such, the exact dynamic range with respect to intracellular concentrations of the actual ligands is still unknown. Future studies might compare the biosensor responses to intracellular concentrations by liquid chromatography mass spectrometry (LC-MS) evaluation of cell lysates or performing assays in cell free conditions. These biosensors are not expected to be quantitative bioassay replacements for analytical techniques such as HPLC or LC-MS, but they are well suited to high throughput screening of adaptive laboratory evolution populations or mutation libraries, where the fluorescence response correlates to the production rate or intracellular pool of the metabolites. In such applications, the biosensors are used for initial screening, while the actual performance of the selected subset of strains is validated by HPLC. Further optimization of the biosensors is possible by targeted modifications to the promoter sequence or to the TF proteins, but this study highlights the utility of these biosensors and that their dynamic ranges are such as to respond to some biologically relevant perturbations such as metabolic knockouts or enzyme activity changes upstream or downstream of ccMA and chorismate nodes.

Dual sensing for metabolites using genetically encoded biosensors in *C. glutamicum* was demonstrated in this study. We showed that by feeding different precursors for ccMA and chorismate (i.e., benzoate and quinate, respectively) to biosensor strains of *C. glutamicum*, green and red fluorescence signals could be used to detect intracellular ccMA and chorismate, respectively. We also observed an indirect effect of quinate (chorismate precursor) on the ccMA biosensor. Given the lack of PCA decarboxylase (aroY) in *C. glutamicum*, it is unlikely that quinate was being shunted through PCA and into ccMA production, especially considering that the ccMA biosensor does not respond to exogenous PCA (Fig. 2c). A strain with gene knockout (Δ*qsuB*) that disconnects the shikimate pathway from the β-ketoadipate pathway did not show the indirect effect of quinate on the ccMA biosensor (Fig. 4f). At this stage, we could not conclude if the effect was due to any metabolic advantage conferred by quinate or any regulatory crosstalk between the shikimate/quinate pathway intermediate and β-ketoadipate pathway.

In summary, we built promoter libraries in *C. glutamicum* ATCC 13032 and screened them to develop and optimize biosensors for a central intermediate (chorismate) and an industrial chemical precursor (ccMA). In the process, we observed what we believe is ccMA transported into *C. glutamicum* ATCC 13032. This work will expand the toolkit for *C. glutamicum*, which is recognized as a promising microbial chassis for bio-based production of a wide range of chemicals, fuels, polymer precursors and healthcare products (Becker, Rohles, et al., 2018).

## Supplementary Material

kuae024_Supplemental_File
