# Supplementary material for: Biosensors for the detection of chorismate and cis,cis-muconic acid in Corynebacterium glutamicum
Source: J Ind Microbiol Biotechnol. 2024 Jun 29;51:kuae024. doi: 10.1093/jimb/kuae024 (PMC11258901; doi:10.1093/jimb/kuae024)
Supplement: kuae024_Supplemental_File [file kuae024_supplemental_file.docx]

**Supporting Information**

**Biosensors for the detection of chorismate and *cis*,*cis*-muconic acid in *Corynebacterium glutamicum***

Jeanette C. Velasquez-Guzman^1,2,5†^, Herbert M. Huttanus^1,5†^, Demosthenes P. Morales^3^, Tara S. Werner^1,5^, Austin L. Carroll^4,5^, Adam M. Guss^4,5^, Chris M. Yeager^2,5^, Taraka Dale^1,5^*, Ramesh K. Jha^1,5^*,

^1^Bioscience Division, Los Alamos National Laboratory, Los Alamos, NM, United States

²Chemistry Division, Los Alamos National Laboratory, Los Alamos, NM, United States

^3^Center for Integrated Nanotechnologies, Los Alamos National Laboratory, Los Alamos, NM, United States

^4^Biosciences Division, Oak Ridge National Laboratory, Oak Ridge, TN, United States

^5^Agile BioFoundry, Emeryville, CA, United States

^†^: equal contribution

*: corresponding author [rjha@lanl.gov](mailto:rjha@lanl.gov), [tdale@lanl.gov](mailto:tdale@lanl.gov)

**Supporting Information 1. Figures**


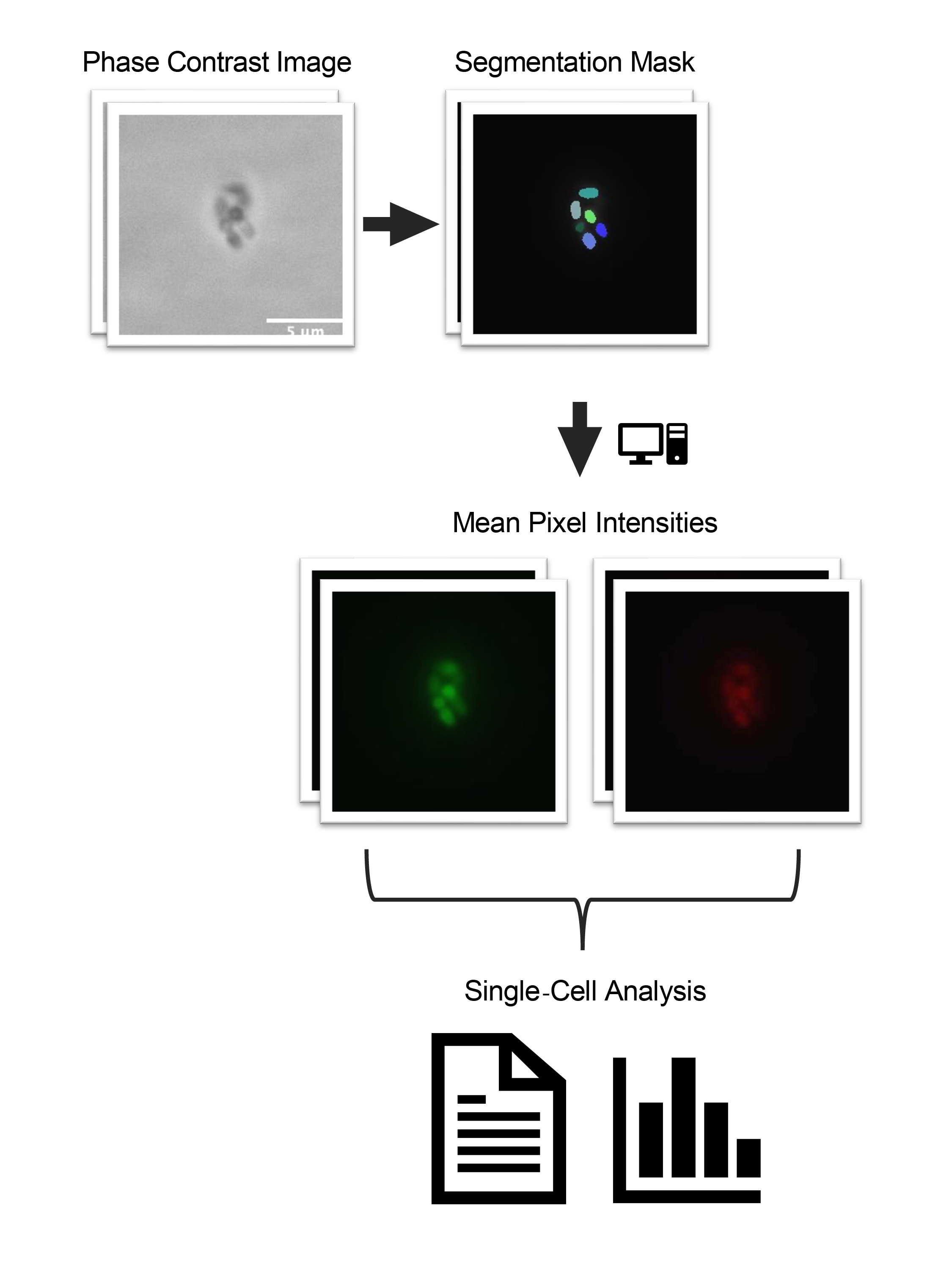


**Figure S1. Illustration of workflow for single-cell analysis by optical microscopy methods.** First a segmentation mask is manually generated against the phase microscope image using Photoshop. A custom Python script is then employed to identify contours and segmenting single-cells (individual colors in mask). Boundaries of individual cells are then used to determine the mean pixel intensity for each cell in each fluorescence channel (green or red). Data points from single-cell analysis are then used for comparisons.

**Figure S2**. (A) Plasmid map for ccMA biosensor constructs (pCG_CatM_C2 and pRJ2010) for *C. glutamicum* 13032. (B) P*_cat_* promoter with features and nucleotides in red that were optimized for biosensor activity as described previously^1^ to make the plasmid library pCg_CatM_promo_Lib1.

**Figure S3. Activity of a *P. putida* optimized ccMA biosensor in *C. glutamicum* 13032 (Δ*catB*).** (A) Biosensor response to addition of benzoate (ccMA precursor) or protocatechuate (PCA, negative control) measured using a BD Accuri C6 Plus flow cytometer. (B) Overlay of fluorescence histograms at increasing benzoate concentration as described in (A).

**Figure S4. ccMA sensing in *P. putida* KT2440 and *C. glutamicum* 13032*.*** The *catB* gene was deleted in both strains to eliminate ccMA catabolism and were transformed with optimized biosensors for ccMA (pCatM_C2 for *P. putida* KT2440^1^ and pRJ2010 for *C. glutamicum* 13032). 1 mM ccMA was supplemented exogenously. The cell population shifts to higher GFP fluorescence only in the case of *C. glutamicum. P. putida* showed a strong biosensor response in the presence of ccMA only when a transporter MucK (ported from *A. baylyi ADP1*) was engineered into it.^2^

**Figure S5. QsuR transcription factor as a basis of chorismate sensor design in *C. glutamicum* 13032.** (A) Native *qsuR* gene (CGL_RS02135) and the downstream regulated gene *qsuA* (CGL_RS02140) encoding a major facilitator superfamily (MFS) transporter. (B) Plasmid construct for chorismate biosensor constructs such as, pJV1 and pJV5E.2. (C) Identified features in P*_qsu_* promoter region and the regions (marked in orange) that were completely or partially randomized using degenerate nucleotides. The degenerate base “N” stands for A/T/C/G, “Y” for C/T, “W” for A/T, “R” for A/G and “M” stands for A/C.

**Figure S6.** Plasmid construct pJV9 consisting of the chorismate biosensor cassette and *ubiC* gene under a weak P*_dapA_* promoter for *C. glutamicum* 13032. Construct pJV10 has the *ubiC-C22* gene in place of wild type *ubiC*.

**Figure S7. A** ccMA/chorismate dual biosensor construct (pJV8) for *C. glutamicum* 13032.

**Figure S8. Time course of ccMA/chorismate dual biosensor plasmid (pJV8) in *C. glutamicum* strain.** JV7 strain (*C. glutamicum* Δ*catB* harboring pJV8) showing green fluorescence (ccMA biosensor response) in the presence of quinate, benzoate or both. The ligand concentrations were 3 mM of quinate, 3 mM of benzoate or 3 mM each of quinate and benzoate. Fluorescence data were collected in a SynergyTM H4 Hybrid Multi-Mode Microplate reader and normalized with the OD_600_ for each timepoint.

**Figure S9.** ccMA biosensor response in strain JV8 consisting of ccMA/chorismate dual biosensor construct (pJV8) in *C. glutamicum* 13032 (Δ*catB*) strain at 10 mM benzoate (ccMA precursor) and variable concentrations of quinate.

**Figure S10. Muconate biosensor response (RJ95A) to benzoate and quinat**e. Though quinate cannot be converted to ccMA in strain RJ95A since it lacks protocatechuate decarboxylase (AroY),^3^ an increase in ccMA biosensor response was observed when quinate (3 mM) and benzoate (3 mM) were added together relative to adding benzoate alone.

**Figure S11. Visualizing cellular morphology and fluorescence response of a dual biosensor in *C. glutamicum* 13032.** JV7 cells (*C. glutamicum*13032 Δ*catB*) were grown with chorismate and/or ccMA precursors, such as quinate and benzoate respectively. (A) Fluorescence micrographs of JV7 cell biosensors with respective treatment with 3 mM quinate, benzoate or combination of the two. Merged image overlays Phase, Green and Red images with correlated pixels presenting "Yellow". Scale bar is 5 microns. (B) Mean pixel intensity (MPI) histogram of cells treated with respective precursor (filled bars) plotted with untreated cells (unfilled bars). P values of <0.001 represent significant differences in means at a 99.5 % confidence interval using a two-tailed Student's t-test comparing the means of treated versus untreated for the corresponding fluorescence channel (green or red). (C) Boxplot comparison of the ratio of MPI between green and red fluorescence for each treatment against the biosensor. Quinate shows little to no difference in profile from the uninduced cells (P=0.341, at a 95 % CI), while benzoate treated cells are distinctly identifiable from uninduced and other treatments (P<0.001, at 95 % CI, Quinate/Benzoate).


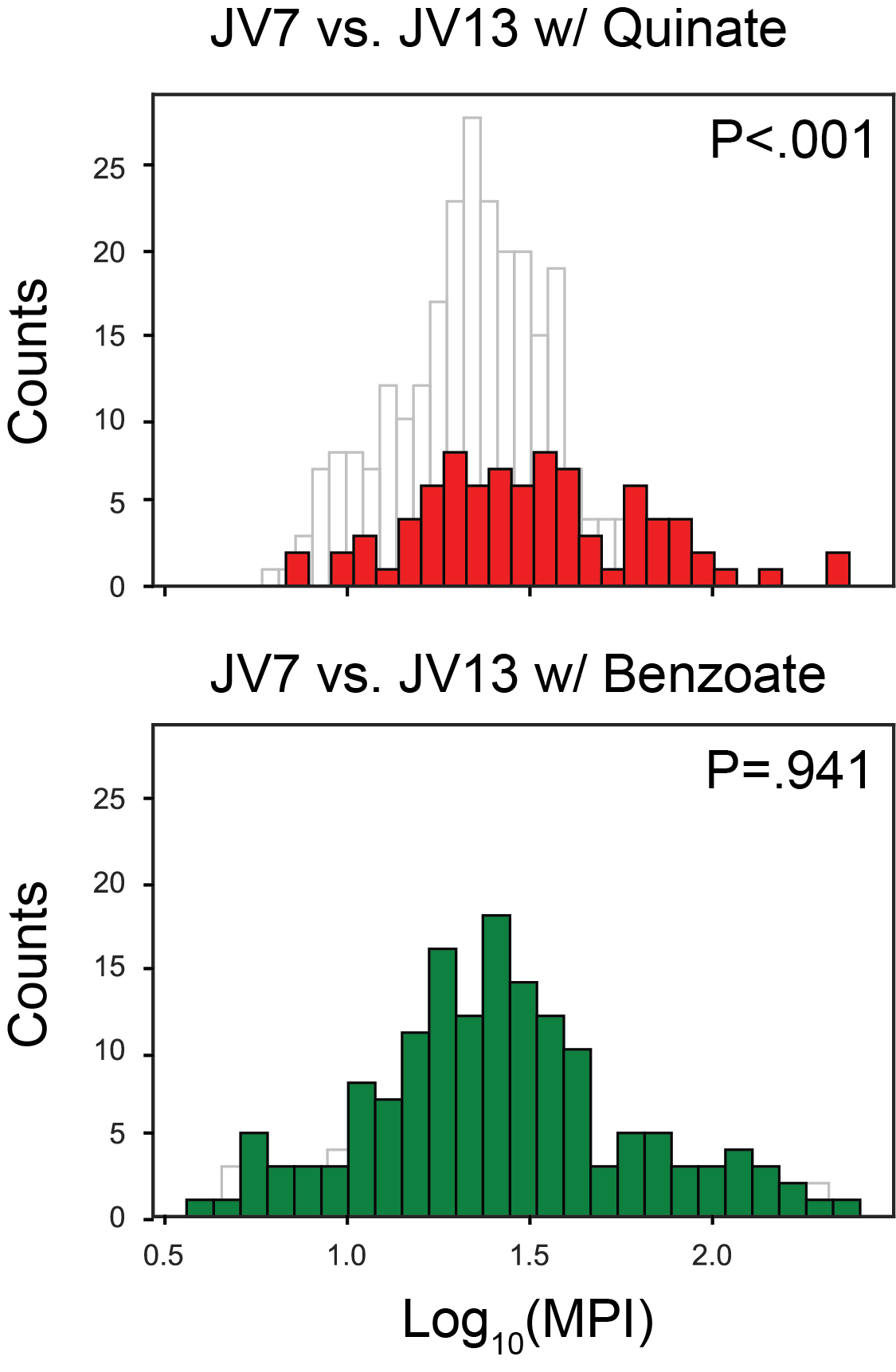


**Figure S12. Comparison of JV7 (open bars) and JV13 (colored bars) dual biosensor response in the presence of quinate or benzoate.** (Top panel) JV13 (*C. glutamicum*13032 Δ*catB* Δ*qsuB)* biosensor demonstrated greater signal intensity in red fluorescence for quinate compared to the JV7 (*C. glutamicum*13032 Δ*catB*) biosensor (P<0.001, for a one-tailed t-test at 99.5 % confidence interval). (Bottom panel) Both biosensors presented similar green fluorescence response to the benzoate feeding (P=0.941, two-tailed t-test at 99.5 % confidence interval). Quinate and benzoate were supplemented in the growth media at a concentration of 3 mM.

**Figure S13.** A temperature sensitive (ts) plasmid construct containing the ccMA biosensor cassette**.** The backbone pMB1/bla was used to insert RepA_ts (RepA replicase with a Pro 🡪 Ser mutation that allows for temperature-dependent replication), apramycin resistance gene marker (ApmR), and β-lactamase gene (AmpR). The ccMA biosensor cassette consisting of *catM*, P*_cat_-opt-2* and *sfgfp* sequences were inserted in the vector to create a temperature sensitive ccMA biosensor.

**Supporting Information 2. Tables**

**Table S1**. Oligonucleotides used in the current work

| oRJ298 | Forward primer for amplification of pRJ2010 to construct pJV1 | CCAGGCATCAAATAAAACGAAAGG |
| --- | --- | --- |
| oRJ170 | Reverse primer for amplification of pRJ2010 to construct pJV1 | TTTTTCCTGCCATACTTTGTTTCGG |
| oRJ299 | Forward Primer for PqsuR | GGCCGAAACAAAGTATGGCAGGAAAAAGTGTGCTAAACCTTTCGGCCG |
| oBH009 | Reverse primer for sfCherry/pQsuRlib1 | CTCCTCACCTTTAGACAGCATATGTATATCTCCTTATCGAAGGTTCCAGCAACAG |
| oRJ300 | Reverse primer for sfCherry/dualterm_gstrand1 | CCTTTCGTTTTATTTGATGCCTGGTTATTTATATAATTCATCCATACCACCAGTAGAATG |
| oBH010 | Forward primer for sfcherry | ATGCTGTCTAAAGGTGAGGA |
| oBH013 | Sequencing primer for qsuR promoter rev | CCATGTGCACCTTAAAACGC |
| oBH049 | Forward primer for confirmation of *qsuD* deletion | CGTTGGGCACGCTGTTC |
| oBH050 | Reverse primer for confirmation of *qsuD* deletion | ATCAAACGTCACCGTGAACTTACC |
| oJV1 | Forward primer for pTS-PBL1to amplify catM_sfGFP cassette with BamHI overhangs | TAAGCAGGATCCTTATTCGATGAGTGGCCTGATATGGTG |
| oJV2 | Reverse primer for pTS-PBL1 to amplify catM_sfGFP cassette with BamHI overhangs | TAAGCAGCGGCCGCCAACTTTAGGAAAGAACCAACATC |
| oJV4 | Reverse primer to amplify *soxR* terminator for dual sensor | TGTGTCCAAAACAAAAAGGGTACAAAACTAAAGCGCCACAAGG |
| oJV5 | Forward primer to amplify *tonB* terminator for dual sensor | GCTTGGTCTATAGTGGCTAGGTACAGTCAAAAGCCTCCGGTCG |
| oJV6 | Forward primer for confirmation of *qsuB* deletion | TCTTCACGGAACCCAGTTCG |
| oJV7 | Reverse primer for confirmation of *qsuB* deletion | GGGAGGCCTTCTTCCATTCC |

**Supporting Information 3. Sequences**

- SI 2.1: *A. baylyi* ADP1 MucK transporter protein^4^

>sp|P94131.1|MUCK_ACIAD RecName: Full=cis,cis-muconate transport protein

MYSNNQRSRIGSHTWKIAFLFAFLALLVDGADLMLLSYSLNSIKAEFNLSTVEAGMLGSFTLAGMAIGGI

FGGWACDRFGRVRIVVISILTFSILTCGLGLTQSFIQFGVLRFFASLGLGSLYIACNTLMAEYVPTKYRT

TVLGTLQAGWTVGYIVATLLAGWLIPDHGWRVLFYVAIIPVLMAVLMHFFVPEPAAWQQSRLAPSKQTET

VKTSAFKLIFQDKRNRNMFILWALTAGFLQFGYYGVNNWMPSYLESELGMKFKEMTAYMVGTYTAMILGK

ILAGFMADKLGRRFTYAFGAIGTAIFLPLIVFYNSPDNILYLLVIFGFLYGIPYGVNATYMTESFPTAIR

GTAIGGAYNVGRLGAAIAPATIGFLASGGSIGLGFVVMGAAYFICGVIPALFIKEKQYDPQQS

- SI 2.2: Alignment of the top hit *C. glutamicum* 13032 protein with MucK

>Permeases of the major facilitator superfamily [Corynebacterium glutamicum ATCC 13032]

Sequence ID: BAC00372.1 Length: 448

Range 1: 40 to 433

Score:147 bits(372), Expect:7e-41,

Method:,

Identities:107/400(27%), Positives:179/400(44%), Gaps:36/400(9%)

Query 28 VDGADLMLLSYSLNSIKAEFNLSTVEAGMLGSFTLAGMAIGGIFGGWACDRFGRVRIVVI 87

+D D+ L+S+ + ++ + LS E +LGS GMAIG GG D+ GR ++ +

Sbjct 40 LDAMDVGLISFVMAALATHWGLSPTETSLLGSIGFVGMAIGASLGGLLADKLGRRQVFAL 99

Query 88 SILTFSILTCGLGLTQSFIQFGVLRFFASLGLGSLYIACNTLMAEYVPTKYRTTVLGTLQ 147

S+L + + T L+ S LRF LGLG+ +TL++E+ P K R ++ L+

Sbjct 100 SLLVYGVATGASALSVSLAMLMALRFVVGLGLGAELPVASTLISEFSPRKVRGRMVVILE 159

Query 148 AGWTVGYIVATLLAGWLI--PDHGWRVLFYVAIIPVLMAVLMHFFVPEPAAWQQSR---- 201

A W +G+I+A ++ +++ D+GWR + +P + AV + +PE + + +

Sbjct 160 AFWALGWIMAAIVGTFVVAGSDNGWRWALALGCVPAIYAVYVRLGLPESVRFLEKKGRHD 219

Query 202 --------LAPSKQTETVKTSAFKLIFQD---------------KRNRNMFILWALTAGF 238

+ E A + D KR ++I+W

Sbjct 220 EAEAIVVSFEEAAAAEGKAADATTAVVHDNAAEGSVSIWSAALRKRTVALWIVWFC---- 275

Query 239 LQFGYYGVNNWMPSYLESELGMKFKEMTAYMVGTYTAMILGKILAGFMADKLGRRFTYAF 298

+ YYG W+PS L ++ G + + + A + G +A ++ +K GRR T A

Sbjct 276 INLSYYGAFIWIPSLLVAD-GFTLVKSFQFTLIITLAQLPGYAVAAWLIEKWGRRSTLAT 334

Query 299 GAIGTAIFLPLIVFYNSPDNILYLLVIFGFLYGIPYGVNATYMTESFPTAIRGTAIGGAY 358

+G+AI L N IL + F +G E +PT +RGT G A

Sbjct 335 FLVGSAISAALYGLANVEWQILVAGCLLSFFNLGAWGALYAIGPELYPTNVRGTGTGAAA 394

Query 359 NVGRLGAAIAPATI-GFLASGGSIGLGFVVMGAAYFICGV 397

GR+ + IAP + +A GG I L F + A+ I +

Sbjct 395 GFGRIASIIAPLIVPPVIAFGGPIAL-FALFATAFAIAAI 433

- SI 2.3: MFS transporter from *C. glutamicum* 13032 with high sequence homology to MucK

>BAC00372.1 Permeases of the major facilitator superfamily [*Corynebacterium glutamicum* ATCC 13032]

MSQRVIFSGMNLTRNDRLDRLPVTSKHKKILGGSGIGWALDAMDVGLISFVMAALATHWGLSPTETSLLG

SIGFVGMAIGASLGGLLADKLGRRQVFALSLLVYGVATGASALSVSLAMLMALRFVVGLGLGAELPVAST

LISEFSPRKVRGRMVVILEAFWALGWIMAAIVGTFVVAGSDNGWRWALALGCVPAIYAVYVRLGLPESVR

FLEKKGRHDEAEAIVVSFEEAAAAEGKAADATTAVVHDNAAEGSVSIWSAALRKRTVALWIVWFCINLSY

YGAFIWIPSLLVADGFTLVKSFQFTLIITLAQLPGYAVAAWLIEKWGRRSTLATFLVGSAISAALYGLAN

VEWQILVAGCLLSFFNLGAWGALYAIGPELYPTNVRGTGTGAAAGFGRIASIIAPLIVPPVIAFGGPIAL

FALFATAFAIAAIAAFTLPEQKGKSLAD

- SI 2.4: Proteins from other Corynebacterium strains with high sequence homology to *C. glutamicum* 13032 MFS transporter
  - SI 2.4.1:

>BAF55884.1 hypothetical protein cgR_2864 [Corynebacterium glutamicum R]

MRSAGVAVCVYGFCLSTPTSDFKGKYFVSQRVIFSGMNLTRNDRLDRLPVTSKHKKILGGSGIGWALDAM

DVGLISFVMAALATHWGLSPTETSLLGSIGFVGMAIGASLGGLLADKLGRRQVFALSLLVYGVATGASAL

SVSLAMLMALRFVVGLGLGAELPVASTLISEFSPRKVRGRMVVILEAFWALGWIMAAIIGTFVVAGSDNG

WRWALALGCVPAIYAVYVRLGLPESVRFLEKKGRHDEAEAIVVSFEDAAAAEGKAVDDTTAVVHDNAAEG

SVSIWSAALRKRTVALWIVWFCINLSYYGAFIWIPSLLVADGFTLVKSFQFTLIITLAQLPGYAVAAWLI

EKWGRRSTLATFLVGSAISAALYGLANVEWQILVAGCLLSFFNLGAWGALYAIGPELYPTNVRGTGTGAA

AGFGRIASIIAPLIVPPVIAFGGPIALFALFATAFAIAAIAAFTLPEQKGKSLAD

- - SI 2.4.2:

>WP_047263143.1 MFS transporter [*Corynebacterium mustelae*]

MNTNSGIELSRNQRLDRLPLTGKHKRMLVGSGVGWALDAMDVGLISFIMAALVTHWGLSPTETSWLAAIG

FVGMAVGATFGGLLADKFGRRQIFAATLLVYGLATGASALAGGLATLMVLRFIVGLGLGAELPVASTLIS

EFSPRAIRGRMVVALEAFWALGWIMAAIIGRFVVTTGHDGWRWALALGCVPALYALFVRLKLPESVRFLE

AKGRHAEAEKIVAGFESELSVKQLSALNATTSTPPPNDSMDSAVSLWSKSFRRRTAILWIIWFCMNLGYY

GAFIWIPSLLVADGFSLVKSFQFTLIITLAQLPGYAVAAWLIEVWGRRATLATFLVGSALAAAGYGFAGS

EATIIVAGCLLSFFNLGAWGALYAISPELYPTEIRGRGTGAAAGFGRIASIMAPLIVPPLISVGGTSALF

SLFGATFICATFASLLLPEQRGKSID

- SI 2.5:

> QsuR sequence (CGL_RS02135)

WP_003855552.1 MULTISPECIES: LysR family transcriptional regulator [Corynebacterium]

MHLNQLEFFIAVAQHGQINRAAEELLISQPALSRQISALEKSVGAPLFERHSRGVSLTKAGEILHEEALR

TLSRMQSVVDEIQSGEHLITSINIGVPPGIPIDWLRCQLIDLGPETRISLIESPTDDQLKLLKQRELDIA

LCRRQSEAFATTLVHEQELGIVVRKNSELHQKVAGKDNATLFDLEGLRVLAHSRGEVRIQEEILKNAMLA

AGVNATWIFRKFGQYSSLIADLVQADVALTTEESARTNFPSWQWVPIEGEDASGNDLVVRTWITWNPQPT

PAVKALIQKFIDGN

- SI 2.6: *C. glutamicum* reference genome

NC_003450.3

- SI 2.7: *qsuR-qsuA* with intergenic region

CGL_RS02135 (WP_003855552.1):CGL_RS02140 (WP_011013638.1) with intergenic region (P*_qsu_*_)_

441598-444161

>NC_003450.3:441598-444161 Corynebacterium glutamicum ATCC 13032, complete sequence

TCAGTTTCCGTCAATAAATTTCTGGATCAGGGCCTTCACCGCGGGGGTGGGTTGGGGGTTCCAGGTGATC

CAGGTGCGAACAACAAGGTCATTTCCGGAGGCGTCTTCGCCTTCGATGGGGACCCATTGCCAGCTGGGGA

AGTTGGTGCGGGCGGATTCCTCTGTTGTGAGTGCGACATCGGCCTGGACAAGGTCTGCGATCAGTGAGCT

ATATTGCCCAAATTTTCGGAAGATCCACGTGGCATTAACTCCTGCGGCGAGCATGGCGTTTTTGAGGATT

TCTTCCTGAATTCTTACTTCACCGCGGGAGTGTGCGAGGACTCGAAGCCCTTCAAGATCGAAGAGTGTGG

CGTTGTCTTTTCCTGCGACTTTTTGGTGCAGTTCGGAGTTTTTTCGGACGACGATTCCCAGTTCTTGTTC

GTGGACAAGTGTGGTGGCAAAGGCCTCGCTTTGGCGTCGACAAAGGGCGATGTCGAGTTCGCGTTGTTTA

AGAAGTTTTAGCTGATCATCGGTGGGGGATTCGATCAGTGAAATGCGGGTCTCGGGGCCTAAATCGATGA

GTTGGCAGCGCAACCAGTCGATGGGGATTCCAGGGGGAACTCCGATGTTGATGCTGGTGATGAGGTGCTC

ACCGGATTGGATTTCATCGACTACCGATTGCATCCTGCTAAGCGTTCGGAGGGCTTCTTCGTGGAGGATT

TCTCCGGCCTTTGTGAGGGAGACACCGCGGGAATGGCGTTCGAAGAGTGGAGCTCCGACGGATTTTTCAA

GTGCGGAGATCTGTCGGCTGAGAGCGGGTTGGGAAATGAGGAGTTCTTCGGCGGCGCGGTTGATCTGTCC

GTGTTGGGCTACTGCGATGAAAAATTCGAGCTGATTGAGGTGCAT**GTGTGCTAAACCTTTCGGCCGGTAT**

**GCAAGAGATGGATATCACGGTTAATGTTTATGCATTTGTACTATAGATCACAATTGCTTAGAGTTCAGGG**

**AAAGGGTCATACATCCACCCCTTCTGAGAAGTTAAAAGTAGGGACGCCAACACAATTGGCAGAATTTAGA**

**ACTTCAATGCATTACCCACAATTTCAATCGGTTTATACAACCAGCCTCTAACTGGCAACAGGACTGCAGA**

**CAGAAACTGTTGCTGGAACCTTCGATGAACAGGATCGACA***ATGAGCGAACAACTTCAGGGTGTAACTCAC*

*TCCGAATCAACTCCGGGCAAGACGCCCAAGCGAGCAGCACTATCCAGCTGGATCGGCTCAGCTCTCGAAT*

*ACTACGACTTCGCTGTTTACGGAACCGCTGCAGCGCTGGTTCTTAACCACCTCTTCTTCCCAGCTGATAC*

*TTCACCAGGCATCGCAATTTTGGCTGCGATGGGTACCGTGGGTGTTGCTTATGTGGTTCGCCCTCTTGGT*

*GCGCTGATCATGGGTCCATTAGGTGACCGTTACGGACGTAAATTTGTCCTCATGCTGTGCCTCTTCCTGA*

*TTGGAGCATCCACTTTCGCAGTTGGCTGCTTGCCAACATTTGATCAGGTCGGTTACTTGGCTCCGGCACT*

*GTTGGTGCTGTGCCGTGTGATCCAGGGACTGTCTGCATCCGGTGAGCAGTCCAGTGCGATTTCCGTTTCT*

*TTGGAGCACGCCGATGAGCGTCACCGCGCATTTACTGCTAGCTGGACTCTTCACGGAACCCAGTTCGGTA*

*CCTTGCTGGCAACCGGAGTATTTATCCCATTCACCTTGTTCCTGAGTGAAGATGCTCTAATGTCATGGGG*

*TTGGCGCGTTCCGTTCTGGCTGTCCGCTGCTGTTGTTTTGGTTGCTTTCCTCATCCGTCGTGGACTGGAA*

*GAGCCACCAGCATTCCGTGAAAACAAGGAAGCAGTTGCAGGCGCAGCATCTCCACTGGCGATGACCTTGC*

*GTTACCACAAGGCGGCGGTTGCTCGCGTTGCTATTGCTGCGATGATCAACTCCGTGAACATTGTGTTTAC*

*TGTGTGGGCACTGTCGTTCGCCACCAACATTGTTGGCCTGGATCGTTCAACTGTTTTGCTGGTTCCAGTT*

*GTTGCGAACTTGGTTGCACTGATTGCGATTCCTTTGTCCGGCATGCTGGCTGACCGCATTGGTCGCCGAC*

*CAGTGTTCATCATGGGTGCCATTGGTGGTGGCCTGGCCATGAACGGTTACCTGGGAGCTATCTACTCCGG*

*CAATTGGACCATGATCTTCTTCATGGGCGTGTTGATGTCTGGTCTGCTGTACTCCATGGGTAATGCCGTG*

*TGGCCAGCGTTCTACGCAGAAATGTTCCCAACCTCTGTGCGTGTCACCGGCTTGGCTCTTGGAACTCAGA*

*TTGGTTTCGCAGTCTCTGGTGGTTTCGTCCCAGTTATCGCATCCGCGCTTGCTGGTGATCAGGGTGACCA*

*GTGGATGAAGGTGTCCATCTTCGTTGGTGTTGTTTGTGTGATTTCTGCACTGGTTGCCATGACCGCTAAG*

*GAAACCAAGGCTCTGACTCTGGATGAGATCGATGCTCTGCACACTGCTGGTGGTGAGGCCGCAGACCTGG*

*CAGCCGCAAGCAAAGCCTCCGAGGCCCAACTCGCGGCTCAGTAA*

CGL_RS02135 (*qsuR*): Underlined

Intergenic region: **Bold**

CGL_RS02140 (MFS transporter): *Italics*

**References**

(1) Bentley, G. J.; Narayanan, N.; Jha, R. K.; Salvachúa, D.; Elmore, J. R.; Peabody, G. L.; Black, B. A.; Ramirez, K.; De Capite, A.; Michener, W. E.; Werner, A. Z.; Klingeman, D. M.; Schindel, H. S.; Nelson, R.; Foust, L.; Guss, A. M.; Dale, T.; Johnson, C. W.; Beckham, G. T. Engineering Glucose Metabolism for Enhanced Muconic Acid Production in *Pseudomonas Putida* KT2440. *Metabolic Engineering* **2020**, *59*, 64–75. https://doi.org/10.1016/j.ymben.2020.01.001.

(2) Shin, S.-M.; Jha, R. K.; Dale, T. Tackling the Catch-22 Situation of Optimizing a Sensor and a Transporter System in a Whole-Cell Microbial Biosensor Design for an Anthropogenic Small Molecule. *ACS Synth. Biol.* **2022**, *11* (12), 3996–4008. https://doi.org/10.1021/acssynbio.2c00364.

(3) Lee, H.-N.; Shin, W.-S.; Seo, S.-Y.; Choi, S.-S.; Song, J.; Kim, J.; Park, J.-H.; Lee, D.; Kim, S. Y.; Lee, S. J.; Chun, G.-T.; Kim, E.-S. Corynebacterium Cell Factory Design and Culture Process Optimization for Muconic Acid Biosynthesis. *Sci Rep* **2018**, *8* (1), 1–12. https://doi.org/10.1038/s41598-018-36320-4.

(4) Williams, P. A.; Shaw, L. E. MucK, a Gene in Acinetobacter Calcoaceticus ADP1 (BD413), Encodes the Ability to Grow on Exogenous Cis,Cis-Muconate as the Sole Carbon Source. *J Bacteriol* **1997**, *179* (18), 5935–5942. https://doi.org/10.1128/jb.179.18.5935-5942.1997.
